# Effects of facilitated family case conferencing for advanced dementia: A cluster randomised clinical trial

**DOI:** 10.1371/journal.pone.0181020

**Published:** 2017-08-07

**Authors:** Meera Agar, Tim Luckett, Georgina Luscombe, Jane Phillips, Elizabeth Beattie, Dimity Pond, Geoffrey Mitchell, Patricia M. Davidson, Janet Cook, Deborah Brooks, Jennifer Houltram, Stephen Goodall, Lynnette Chenoweth

**Affiliations:** 1 Faculty of Health, University of Technology Sydney, Ultimo, New South Wales (NSW), Australia; 2 South Western Sydney Clinical School, University of New South Wales, Liverpool, NSW, Australia; 3 Ingham Institute for Applied Medical Research, Liverpool, NSW, Australia; 4 Improving Palliative Care through Clinical Trials (ImPaCCT), Sydney, NSW, Australia; 5 Sydney Medical School, The University of Sydney, Ultimo, NSW, Australia; 6 School of Nursing, Queensland University of Technology, Herston, Queensland (QLD), Australia; 7 School of Medicine and Public Health, The University of Newcastle, Newcastle, NSW, Australia; 8 Faculty of Medicine, The University of Queensland, St Lucia, QLD, Australia; 9 School of Nursing, Johns Hopkins University, Baltimore, Maryland, United States of America; 10 Centre for Health Research and Evaluation (CHERE), Faculty of Business, UTS, Haymarket, NSW, Australia; 11 Centre for Healthy Brain Ageing, University of New South Wales, Randwick, NSW, Australia; University of Glasgow, UNITED KINGDOM

## Abstract

**Background:**

Palliative care planning for nursing home residents with advanced dementia is often suboptimal. This study compared effects of facilitated case conferencing (FCC) with usual care (UC) on end-of-life care.

**Methods:**

A two arm parallel cluster randomised controlled trial was conducted. The sample included people with advanced dementia from 20 Australian nursing homes and their families and professional caregivers. In each intervention nursing home (n = 10), Palliative Care Planning Coordinators (PCPCs) facilitated family case conferences and trained staff in person-centred palliative care for 16 hours per week over 18 months. The primary outcome was family-rated quality of end-of-life care (End-of-Life Dementia [EOLD] Scales). Secondary outcomes included nurse-rated EOLD scales, resident quality of life (Quality of Life in Late-stage Dementia [QUALID]) and quality of care over the last month of life (pharmacological/non-pharmacological palliative strategies, hospitalization or inappropriate interventions).

**Results:**

Two-hundred-eighty-six people with advanced dementia took part but only 131 died (64 in UC and 67 in FCC which was fewer than anticipated), rendering the primary analysis under-powered with no group effect seen in EOLD scales. Significant differences in pharmacological (*P* < 0.01) and non-pharmacological (*P* < 0.05) palliative management in last month of life were seen. Intercurrent illness was associated with lower family-rated EOLD Satisfaction with Care (coefficient 2.97, *P* < 0.05) and lower staff-rated EOLD Comfort Assessment with Dying (coefficient 4.37, *P* < 0.01). Per protocol analyses showed positive relationships between EOLD and staff hours to bed ratios, proportion of residents with dementia and staff attitudes.

**Conclusion:**

FCC facilitates a palliative approach to care. Future trials of case conferencing should consider outcomes and processes regarding decision making and planning for anticipated events and acute illness.

**Trial registration:**

Australian New Zealand Clinical Trial Registry ACTRN12612001164886

## Background

Care in advanced dementia requires a palliative approach focused on quality of life (QOL) [1–4]. Nursing home residents with advanced dementia often receive suboptimal palliation due to limited staff awareness and training [2, 5–14]. Communication between staff, health services and families (including surrogate decision-makers) addressing palliative care needs is often poor [6, 7]. Decisions include whether hospital transfers and acute interventions such as intravenous antibiotics will offer net benefit versus detriment to QOL [8, 9, 12, 15–18]. Surrogates should be involved in symptom management decisions and care planning, and provide the personal context [7, 10, 14].

Case conferencing brings together health professionals and other surrogate decision-makers to build consensus on goals of care and appropriate steps for current management and advance care planning. Case conferencing shows promise for improving symptom management in people with dementia living in nursing homes but systematic reviews have highlighted methodological weaknesses in the evidence base [19, 20]. Evidence from randomised controlled trials (RCTs) in the community palliative care setting is more compelling with better maintenance of physical and mental health [21] and decreased hospitalization seen, [22] provided the process is facilitated by appropriate training and organizational support [23–25].

The current study compared the efficacy of facilitated case conferencing versus usual care in improving end of life (EOL) care for persons with advanced dementia living in nursing homes.

## Methods

### Study design

A cluster RCT design was used because the intervention aimed to change the approach to dementia palliative care across nursing homes and randomizing individual residents or staff members would have resulted in contamination. The parallel cluster RCT design is described in a published protocol [26] (see also S1 File), and was conducted over a predefined study period of 18 months between February 2013 to December 2014. Human Research Ethics Committees of the University of NSW, University of Technology Sydney, Queensland University of Technology approved the trial. This paper focuses on the study’s first aim, namely to compare the efficacy of a facilitated approach to family case conferencing with usual care. As such, it focuses on data relevant to evaluating quality of end of life (EOL) care for participants who died during the study period. Data relevant to understanding processes influencing implementation and evaluating cost-effectiveness will be reported in other papers. Details are reported according to the Consolidated Standards of Reporting Trials Checklist for Cluster Randomised Controlled Trials (see S1 Table).

### Randomization and blinding

The study’s statistician (GL) generated a block randomization schedule using a computer-generated allocation sequence, and allocated nursing homes to each arm after collection of institutional-level baseline data, stratifying by organizational affiliation. Due to the system-level nature of the intervention, participating investigators, project managers and nursing home managers could not be blinded to the evaluative aim of the research or to nursing home allocation. Staff, residents and families at each nursing home were blinded to the evaluative aim of the study, but those in nursing homes allocated to the intervention arm were aware of associated changes to practice (see below). Research staff were blinded to the evaluative aim of the study and collected data from nursing homes in only one arm to reduce the likelihood they would notice systematic differences in practice.

### Sites

Sites were 20 nursing homes in two major Australian cities meeting the following criteria: 1) ≥100 beds, 2) ≥50% people with dementia, and 3) designated as facility providing intensive level of nursing home care. Nursing homes were identified from an Australian government website list [27] and approached in alphabetical order to minimise selection bias.

### Participants

The MORECare initiative[28] informed target participants, namely people with advanced dementia living in residential care (‘residents’) where surrogate decision-maker involvement for palliative care planning is needed. Potentially eligible residents were identified by nursing home managers and screened by the study team. Residents needed to have dementia documented in nursing home records and advanced dementia as determined by scores on the: 1) Functional Assessment Staging Tool (FAST)[29] in dementia (≥6a, stable for 1 month), and 2) Australia–modified Karnofsky Performance Status (AKPS) [30] ≤50. These criteria were chosen because a FAST stage 7c combined with functional dependency (measured here by the AKPS) is predictive of an average survival of <6 months, and the study’s primary endpoint focused on end of life care [31]. A family member with legal authority provided written informed consent on behalf of the resident. Either the same or another family member involved in making decisions about the resident’s care also gave written informed consent for their own participation in the study. Written informed consent was collected after randomization of nursing homes.

All permanent registered and enrolled nurses and care assistants of participating nursing homes were invited to join the study via staff meeting presentations and email circulars and gave informed consent after randomization of nursing homes.

### Intervention

Facilitated case conferencing (FCC) was compared with usual care (UC) as follows.

#### Facilitated case conferencing (FCC)

Theoretical frameworks underpinning FCC included the expected trajectory of advanced dementia [2] and evidence-based strategies for organizational culture change (clinical leadership [32] and train-the-trainer[33]). A Registered Nurse was trained as a Palliative Care Planning Coordinator (PCPC) in each nursing home working for 2 days per week or equivalent to: 1) identify residents with advanced dementia likely to benefit from a case conference; 2) organise, set an agenda, chair and document case conferences with optimal participation by family, multi-disciplinary nursing home staff and external health professionals (e.g. General Practitioner’s (GP’s)); 3) develop and oversee implementation of palliative care plans; and 4) train nursing and direct care staff in person-centred palliative care. The key features of the case conference model were: use of pre-defined specific clinical triggers for a case conference; used a shared agenda setting model where the resident, their family and all multidisciplinary staff could specify a priori areas for discussion; required attendance of the resident and/or their substitute decision maker or family member(s); was facilitated by the PCPC to ensure optimal participation of attendees; and was followed by a communication strategy to summarise actions and plan arising from the case conference. Discussion topics were not limited and were individualised to what was seen as important for the resident; and could include care planning, current and future treatment decision making, information sharing, meeting resident preferences or needs and advance care planning.

#### Usual care (UC)

In nursing homes randomised to UC, no staff education, training or support was provided. No restrictions were placed on nursing homes’ education programme, or approach to care planning and decision-making.

### Data collection

Details of baseline, process and outcome data are provided in the published protocol [26]. Resident-level measures used validated measures in advanced dementia which perform well with surrogate report. Data on resident and nursing home variables likely to influence EOL care [34], and FCC fidelity was also collected. All nurse and family-rated measures were collected via face-to-face or telephone interview with the research team.

The primary outcome was family-rated EOL care (End of Life in Dementia (EOLD) Scales)[35] optimally assessed four to six weeks following the residents death: a) symptom-related comfort during the last 7 days of life (Comfort Assessment in Dying with Dementia; CAD–EOLD); b) symptom management in the last 90 days of life (Symptom Management at the End of Life in Dementia; SM–EOLD); and c) family or caregiver’s satisfaction with care during the last 90 days of life (Satisfaction With Care at the End of Life in Dementia; SWC–EOLD). Resident-level secondary outcomes were nurse-rated CAD-EOLD and SM-EOLD ratings as soon as possible following the resident’s death; nurse-rated QOL three monthly (Quality of Life in Late-stage Dementia (QUALID) Scale [36]), and symptoms and care in the last month of life extracted from nursing home and medical records (symptom control medication [e.g. analgesics] versus diagnosis oriented [e.g. antibiotics, anti-epileptics, anxiolytics, steroids] commencement, cessation and dose alteration; non-pharmacological strategies [e.g. family attendance, body positioning]; symptom assessment frequency; acute care episodes and potentially inappropriate non-palliative interventions [ventilation, resuscitation, enteral feeding, intravenous antibiotics and fluids, dialysis, transfusion, oxygen and surgery). Comorbidities were collected from the resident record classified as life threatening (e.g. cancer, organ failure, neurological), disabling (e.g. hearing/vision, musculoskeletal) or inter-current acute (e.g. infections).

Nursing home variables were: staff attitudes to, knowledge of and confidence in providing palliative care to people with advanced dementia (Palliative Care for Advanced Dementia [qPAD] [37]); ratio of nursing staff hours to beds; and proportion of residents with dementia.

Fidelity to protocol (intervention ‘dose’) at the resident level was collected for use in per protocol (PP) analyses, but could not be measured as planned [26] as many UC nursing homes did not routinely collect detailed information about case conferences (e.g. triggers, attendance, issues discussed), and encouraging this data collection may have led to contamination. A simpler dose measure was used, namely whether or not participating residents received a case conference during their time in the study. Dose at the nursing home level consisted of four indicators concerning the extent to which PCPCs: 1) were able to work 2 days per week, 2) were supported by managers, 3) fulfilled expectations outlined in training, and 4) diffused their role among other staff. Each indicator was scored 0, 1 or 2, with 0 representing a lesser extent, 1 a moderate extent and 2 a large extent.

### Sample size

Assuming an intracluster correlation coefficient (ICC) of 0.05 (estimated from unpublished data sourced from Dutch nursing homes), a sample size of 8 clusters per arm with 15 residents (who died during the study period and for whom EOL outcomes would be available) per cluster (i.e. N = 240 in total), was considered adequate to identify a between-arm difference of 0.5 standard deviation (SD) on the EOLD scale with a two-sided 5% significance level and power of 80%. We conservatively anticipated a 10% resident dropout rate (e.g. withdrawal of consent to participate in the study; resident moved to another nursing home). Allowing for this, a recruitment target of 272 people with advanced dementia (17 per site) was set. This calculation incorporated an estimate that almost all people (98%) meeting the inclusion criteria would die (and so yield data on EOL care) within the study period (<18 months) based on review of dementia- specific mortality data from local nursing homes and evidence from the literature relating to prognostic variables referred to above [31, 38]. In other words, with a 10% withdrawal rate and 2% survival rate, of 272 participants we would expect 27 to withdraw, and of the remaining 245, five to survive to the end of the study period, resulting in a total sample size of 240 available for analysis.

### Analysis

Between-arm differences at baseline in resident and nursing home characteristics and secondary outcomes were analysed using two-tailed t-tests for continuous variables where the distribution was normal or the Mann-Whitney U z-test where distribution was significantly skewed. Chi-square tests were used to determine whether differences were significant on categorical variables.

Analyses of primary and secondary outcomes were on an intention to treat (ITT) basis. Generalised linear mixed models (GLMMs) were used to analyse the impact of the intervention on EOL care (EOLD) and quality of life (QUALID; last measurement during the 90 days prior to resident death). GLMMs were run without adjustment to the dependent variables, and following an evaluation of model best fit using information criteria, were conducted assuming normal distributions with identity links. Residual plots were visually inspected to ensure there were no substantial deviations from normality or homoscedasticity. GLMMs allow for the inclusion of fixed and random effects in the model. These models account for nested sources of variability in data, such as when residents with different characteristics are clustered within nursing homes. In these models, the nursing home was included as a random effect, accounting for intra cluster-correlations in the sample and consequently producing better fixed-effect estimates. While some nursing home level variables were included in the models, the inclusion of a random effect allows for other unmeasured nursing home level effects. In all models, there were resident-level control variables (comorbidities: presence or absence of a disabling condition, an inter-current acute condition, or a life-threatening condition) and nursing home-level control variables (proportion of residents with dementia; baseline ratio of nursing staff hours to bed number; and median staff qPAD Knowledge Test and Attitude Scale scores). These models were rerun in a series of Per Protocol (PP) analyses, excluding nursing homes from the FCC arm that did not implement the intervention to any degree planned (i.e. had a score of ‘0’ on all four nursing home dose variables), and including the resident-level dose variable of whether or not a case conference was received.

Statistical analyses were conducted using IBM SPSS Statistics version 22 (IBM Corp., Armonk, NY). Alpha, the significance level threshold, was set at 0.05 for all statistical tests.

## Results

**Fig 1:**
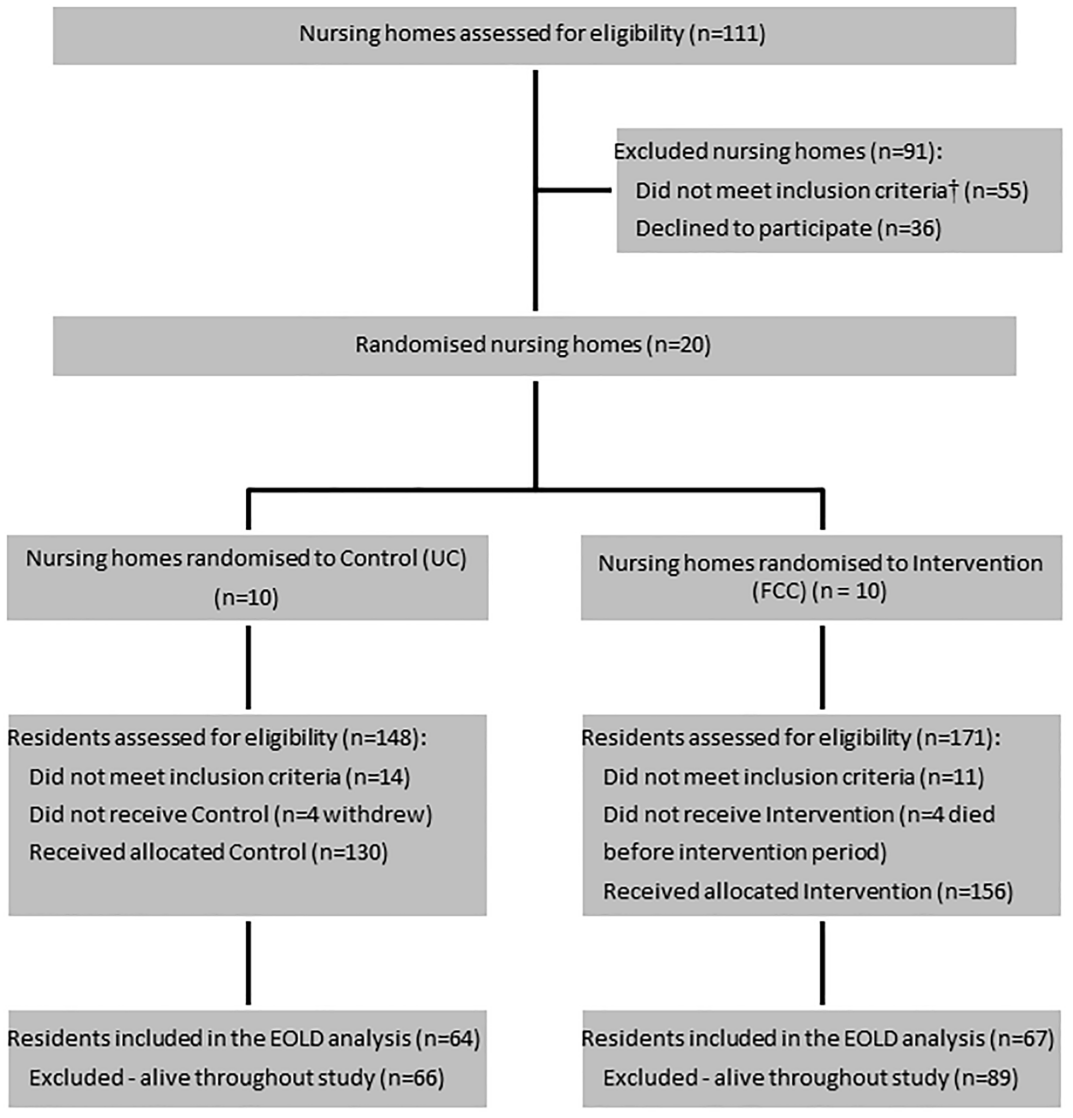
Study flowchart for IDEAL study residents included in EOLD analysis (N = 131). † Original nursing home eligibility criteria were amended, allowing three facilities with bed counts <100 to be included.

Forty-nine nursing homes were approached to recruit the 20 needed (Fig 1). Reasons for declining to participate included a lack of interest in research, other research projects or confidence in their case conferencing and palliative care programs. Due to difficulties recruiting facilities, three facilities were included who had less than 100 beds (two UC with 46 and 88 beds; one FCC with 75 beds). Three hundred and nineteen residents were assessed for eligibility, with 25 not eligible. Informed consent was obtained for 294 residents, and four died prior to the intervention period commencing and four withdrew. The baseline sample comprised 130 residents (UC facilities) and 156 residents (FCC facilities). During the study, 131 (46%) participants died, 64 in UC and 67 in FCC. Median time to death was 7 months (inter-quartile range [IQR] 9), with no difference between the UC and FCC arms (*P* = 0.27).

Baseline characteristics of the 20 nursing homes and 131 people who died during the study are in Table 1. Participants in FCC and UC arms had similar baseline characteristics, though FCC participants were more likely to be born in Australia and were visited less frequently. FCC and UC nursing homes were similar in proportion of people with dementia during the previous 3 months and nursing staff hours to bed ratios. However, nurses and care assistants in the FCC arm had significantly higher baseline qPAD knowledge scores, indicating a greater knowledge of palliative care for advanced dementia.

**Table 1:** Baseline characteristics of nursing homes (N = 20) and participants who died (N = 131).

		Usual Care (n = 64)	FCC (n = 67)	TOTAL (N = 131)
**Nursing home characteristics**				
Proportion of residents with dementia, median (IQR)		67.5 (24)	67.5 (28)	67.5 (24)
Nursing staff hours to bed ratio, median (IQR)		20.7 (8.9)	21.2 (4.2)	21 (5.9)
Questionnaire on Palliative care for Advanced Dementia staff median (IQR)				
	staff Knowledge Test	14 (3)	16 (4)‡	15 (4)
	staff Attitude Scale	48 (8)	48 (8)	48 (8)
**Participants at baseline**				
Age, mean ± SD		85.8 ± 8.2	84.7 ± 7.9	85.3 ± 8.0
range		57–101	60–104	57–104
Female, n (%)		37 (58)	41 (61)	78 (60)
Born in Australia, n (%)		33 (52)	47 (70)†	80 (61)
Length of stay (months), median (IQR)		20.5 (43)	29 (41)	26 (42)
Time to death (months), median (IQR)		8 (8)	6 (10)	7 (9)
Died in nursing home, n (%)		55 (89)	58 (88)	113 (88)
Visitor frequency, n (%)				
	Daily or more	22 (34)	13 (19)†	35 (27)
	Between daily-weekly	27 (42)	15 (22)	42 (32)
	Weekly	6 (9)	19 (28)	25 (19)
	Fortnightly or less	9 (14)	20 (30)	29 (22)
Functional Assessment Staging Tool, n (%)				
	level 6	13 (20)	19 (28)	32 (24)
	level 7	51 (80)	48 (72)	99 (76)
Australian-modified Karnofsky Performance Status, n (%)				
	20: totally bedfast	18 (28)	12 (18)	30 (23)
	30: almost completely bedfast	14 (22)	8 (12)	22 (17)
	40: in bed >50% of the time	10 (16)	20 (30)	30 (23)
	50: considerable assistance	22 (34)	27 (40)	49 (37)
Quality of Life in Late Stage Dementia, median (IQR)		25 (13)	25 (9)	25 (10)

FCC, facilitated case conferencing; IQR, inter-quartile range; SD, standard deviation

† *P* < 0.05

‡ *P* < 0.001

### End of life outcomes

Family-reported EOL outcomes were available for approximately three-quarters of the participants who died (76%, 99/131), with no systematic differences in demographic characteristics for people with or without a family EOLD report. Family-rated EOLD measures were completed at a median 7.3 weeks (IQR 5.7; range 2.7 to 24 weeks) and nurse reports at a median 2.4 weeks (IQR 4.0; range 0 to 22 weeks); Table 2 outlines summary statistics. There was no significant group effect on the EOLD scales in any of the models (i.e. family-rated CAD-EOLD, SM-EOLD and SWC-EOLD or nurse-rated CAD-EOLD and SM-EOLD).

**Table 2:** EOLD scores of nursing homes participants who died (N = 131)†.

	Usual Care (n = 64)	FCC (n = 67)	TOTAL (N = 131)
**Family-rated EOLD care, mean (SD)**			
CAD-EOLD	35.5 (5.9)	34.7 (5.9)	35.1 (5.9)
range (possible 14–42)	18–42	15–42	15–42
SM-EOLD	31.7 (7.4)	29.0 (9.5)	30.3 (8.6)
range (possible 0–45)	13–44	9–45	9–45
SWC-EOLD	30.3 (4.2)	31.0 (5.3)	30.7 (4.8)
range (possible 10–40)	20–40	20–40	20–40
**Nurse-rated EOLD care, mean (SD)**			
CAD-EOLD	33.3 (5.7)	32.1 (6.1)	32.7 (5.9)
range (possible 14–42)	23–42	16–42	16–42
SM-EOLD	23.2 (8.3)	22.4 (9.6)	22.7 (9.0)
range (possible 0–45)	4–40	6–40	4–40

† missing items varied between 7.1 and 16.2% for family-rated EOLD scores and between 1.6% and 8.7% for nurse-rated EOLD scores. CAD-EOLD, Comfort Assessment in Dying with Dementia (higher scores, more comfort); SM-EOLD, Symptom Management at the End of Life in Dementia (higher scores lower symptom frequency); SWC-EOLD, Satisfaction With Care at the End of Life in Dementia (higher scores, greater satisfaction).

ICCs for the CAD-EOLD and SWC-EOLD were less than assumed (0.008 and negative respectively) whereas for SM-EOLD the ICC was greater than assumed (0.124). Staff-reported EOLD were missing for four people. There was no significant group effect on the EOLD scales in any of the models. In the ITT models, presence of inter-current acute comorbidity was associated with lower family rated satisfaction with care (SWC-EOLD) and poorer staff rated comfort level (CAD-EOLD) (see Table 3).

**Table 3:** Generalised linear mixed model predictors of quality of end of life care in dementia (EOLD): Intention to treat models.

	*Family rated* *SWC-EOLD*		*Staff rated* *CAD-EOLD*	
	Coefficient	*F-test*	Coefficient	*F-test*
Arm				
Usual care	.13	.91	1.68	.13
FCC	0		0	
Proportion dementia	-.01	.76	.05	.20
qPAD knowledge	.35	.44	.48	.25
qPAD attitude	.29	.20	-.05	.80
Staff hours to bed ratio	.07	.61	-.02	.87
Comorbidity:				
Disability				
Absent	.80	.46	1.98	.07
Present	0		0	
Inter-current acute				
Absent	**2.97**	**.030†**	**4.37**	**.003‡**
Present	0		0	
Life threatening				
Absent	-.87	.41	-.60	.59
Present	0		0	

FCC, facilitated case conferencing; qPAD, Questionnaire on Palliative care for Advanced Dementia

N.B. ‘F-test’ refers to the *P*-value associated with the F test

† Poorer family rated satisfaction with care in those with inter-current acute comorbidity

‡ Poorer staff rated comfort assessment in dying in those with inter-current acute comorbidity

PP analyses indicated positive associations between family-rated EOLD satisfaction with care and better staff attitudes, increased ratios of nursing staff hours to beds, and lower proportions of people with dementia in the nursing home, in addition to absence of inter-current acute comorbidities (see Table 4). Symptom management was rated as poorer by staff for those participants who had a case conference, regardless of treatment arm. In the FCC arm, 69% (n = 46/67) of residents who died received at least one case conference compared with 44% in the UC arm (n = 28/64) (*P* = 0.004). Staff ratings of CAD-EOLD were higher in nursing homes with higher proportions of people with dementia. CAD-EOLD was also rated as lower in people with a comorbid disability or with an inter-current acute condition.

**Table 4:** Generalised linear mixed model predictors of quality of end of life care in dementia (EOLD): Per protocol models with resident level dose.

	*Family rated* *SWC-EOLD*		*Staff rated* *SM-EOLD*		*Staff rated* *CAD-EOLD*	
	Coefficient	*F-test*	Coefficient	*F-test*	Coefficient	*F-test*
Arm						
Usual care	1.54	.19	-.20	.93	.58	.63
FCC	0		0		0	
Case conference						
Absent	-1.13	.34	**4.37**	**.023₽**	1.78	.14
Present	0		0		0	
Proportion dementia	**-.10**	**.035†**	.07	.41	**.09**	**.050†**
qPAD knowledge	.04	.93	.68	.38	.76	.08
qPAD attitude	**.47**	**.029‡**	.04	.92	-.16	.45
Staff hours to bed ratio	**.52**	**.012‡**	-.03	.93	-.29	.15
Comorbidity:						
Disability						
Absent	1.13	.28	3.22	.06	**2.16**	**.043‡**
Present	0		0		0	
Intercurrent acute						
Absent	**3.31**	**.014‡**	3.50	.12	**4.07**	**.004‡**
Present	0		0		0	
Life threatening						
Absent	-.95	.36	-.83	.62	-.57	.60
Present	0		0		0	

N.B. ‘F-test’ refers to the *P*-value associated with the F test

FCC, facilitated case conferencing; qPAD, Questionnaire on Palliative care for Advanced Dementia

† Better family rated satisfaction with care in nursing homes with lower proportions of residents with dementia; better staff rated comfort assessment in dying in nursing homes with higher proportions of residents with dementia.

‡ Higher family rated satisfaction with care in nursing homes with more positive staff attitudes, more staff hours per bed, and in residents without an inter-current acute comorbidity; higher staff rated comfort assessment in dying in people without a comorbid disability and in residents without an inter-current acute comorbidity

₽ Absence of a case conference was associated with higher staff ratings of symptom management

Ratings of the person’s quality of life (QUALID) during the 90 days prior to death were significantly associated with staff qPAD knowledge in the ITT model, with better quality of life being associated with nursing homes with higher staff knowledge scores (coefficient -1.04, *P* = 0.036). This effect was not observed in the PP model.

### Symptoms and care during the last month of life

The prevalence of symptoms observed to be documented in nursing progress notes during the month prior to death differed between the arms, with the FCC arm significantly more likely than UC to have documentation of pain/discomfort (*P* = 0.04), restlessness (*P* = 0.02), constipation (*P* = 0.002) skin tears (*P* = 0.005) and other symptoms (including being resistive to care, agitation, pressure areas, wounds, respiratory infection and chest rattles) (*P* < 0.001). Symptoms not observed more commonly in one arm versus the other included difficulty swallowing/eating/drinking, drowsiness, breathlessness, coughing, choking/gurgling, vomiting, fear or anxiety, diarrhoea and depression (P > 0.05).

Equal numbers (47%) in FCC (31/66) and UC (n = 29/62) had formal pain assessments, but more documentation of pain assessment frequency was seen in the FCC arm (97% (30/31)) than UC (79% (23/29)). Pain assessments were daily or more often in 40% of the FCC participants (12/30) compared with 17% (n = 4/23) of those in UC (*P* = 0.08).

The majority of participants died in the nursing home in both arms. Pharmacological and non-pharmacological changes to management, and physician input during the month prior to death were significantly more common in the FCC arm (see Table 5). Medication initiations in FCC were more frequently symptom-oriented (e.g. analgesics, laxatives) than diagnosis-oriented (e.g. antibiotics, anti-epileptics, anxiolytics, steroids).

**Table 5:** Care in the last month of life (N = 131).

Care over last month of life	Usual Care (n = 64)	FCC (n = 67)	TOTAL (N = 131)*
Died in nursing home, n (%)	55 (89)	58 (88)	113 (88)
Medication changes, n (%)	45 (75)	58 (94)^₽^	103 (84)
Symptom-oriented, n (%)	19 (42)	48 (83)	67 (51)
Non-symptom-oriented, n (%)	4 (9)	5 (9)	9 (7)
No change, n (%)	22 (49)	5 (9)	27 (21)
Non-pharmacological management, n (%)	42 (68)	56 (85)†	98 (77)
At least one hospital admission, n (%)	11 (18)	13 (19)	24 (19)
Hospital length of stay, median (IQR)	5 (5)	2 (4)	4 (5)
ED presentation without hospital admission, n (%)	6 (10)	6 (9)	12 (9)
Input from health professionals, n (%)	37 (60)	39 (59)	76 (59)
Medical	21 (34)	35 (53)†	56 (44)
Nursing	2 (3)	3 (5)	5 (4)
Allied health	24 (39)	25 (38)	49 (38)
Other	12 (19)	15 (23)	27 (21)
Non-palliative interventions, n (%)	20 (33)	26 (39)	46 (36)

† *P* < 0.05

₽ *P* < .01

* data missing (N ranges from 122 to 129)

ED, emergency department; FCC, facilitated case conferencing; IQR, interquartile range

Non-pharmacological interventions included changes to the physical environment, activities, positioning, family presence, pastoral support, lighting or noise levels, music or move to single room

Non-palliative interventions included: oxygen, subcutaneous fluids, intravenous fluids, intravenous antibiotics, surgery and ‘other’ (e.g. vaccinations; indwelling catheter).

## Discussion

This RCT evaluated EOL outcomes of facilitated family case conferencing in advanced dementia from perspectives of EOL care received (satisfaction) and resident outcomes (comfort, quality of life and quality of dying) [39]. While the study recruited sufficient participants overall, a lower than estimated mortality rate meant the participants with primary outcome data (after death) did not meet target sample size. Although no significant intervention effects were observed on EOL outcomes or QOL, management was more consistent with a palliative approach in FCC.

Higher rates of nurse-documented pain and restlessness, and more frequent pain assessment, which are less readily observed and under-reported symptoms[14], suggest increased awareness of symptoms as a result of FCC. Staff in both arms of the study tended to perceive symptom management as poorer for residents who had a case conference than those who didn’t, suggests case conferences foster proactive identification of symptoms.

Resident and nursing home variables are also informative in understanding factors associated with better EOL care. Inter-current acute comorbidities associated with lower family rating of satisfaction with care and lower nurse-ratings of comfort, highlights the need to plan care not only for expected gradual decline and the last hours to days of life, but also for anticipated acute illness, comorbidities and “crisis events” (e.g. aspiration pneumonia). Case conferences have also been used in nursing homes for other decision-making (e.g. behavioral and psychological symptoms of dementia) [40], and integration of these models is important. Quality of EOL care was associated with proportion of people with dementia, higher nursing and direct care staff hours and attitudes towards advanced dementia, consistent with previous evidence that being cared for in a dementia unit reduces likelihood of hospitalisation [41]. However, it is less clear why these relationships were observed only in the PP analyses or why staff knowledge about advanced dementia was not predictive of EOL care given its association with QOL in the last 90 days of life.

Our study has a number of limitations. The inclusion criteria aimed to capture a group of residents with advanced dementia with a high mortality rate. However, even with a participant population where nearly 40% were totally or completely bedfast (compared to 9% in the largest-scale study to date, the CASCADE [Choices, Attitudes, and Strategies for Care of Advanced Dementia at the End-of-Life] Study [38]) and an 18 -month study period (compared with 12 months in the CASCADE Study), the mortality rate was only 46% (compared with 41% in the CASCADE Study). The local Australian data we further relied on to estimate mortality identified a 50% mortality rate in high level care residents with all-stage dementia, but was not able to distinguish mortality due to dementia versus other comorbid illnesses or limit to those with advanced stage dementia only. We recommend that future studies should use more conservative estimates utilizing published mortality rates. We followed previous trials of case conferencing [42, 43] in taking a pragmatic approach aimed at improving external validity by reflecting the diversity of current practice [44, 45]. While staff, residents and families at each nursing home were blinded to the evaluative aim of the study, those in nursing homes in the intervention arm were aware of the introduction of the PCPC role and changes in case conferencing and staff education and so may have been more inclined to report favourably on the quality of palliative care offered as a result. Whilst we introduced measures to limit sampling bias, it seems likely that participating nursing homes were more proactive in palliative care and quality improvement than the sector average. With the exception of one US [46] and one Dutch [47] study, mean scores for family and nurse ratings on the SWC-EOLD, SM-EOLD and CAD-EOLD across both arms were either comparable with, or higher than, those reported in the international literature [48–54], and hospitalization and potentially non-palliative interventions use were low [12, 43]. It is also possible that we did not control for important confounder, mediator or suppressor variables amongst the myriad resident, family and nursing home factors that may have been influential [55]. Whilst the EOLD Scales have demonstrated responsiveness in previous longitudinal research [56], their focus on end of life care may have lacked sensitivity to complex interactions with nursing home, staff, and family characteristics; missed benefits earlier in the person’s care, or failed to detect the surrogate decision-maker’s perceived support and engagement in decisions and care choices. Also, timing of EOLD scale administration occurred substantially later than the optimal window in many cases, risking a recall bias of unknown direction and similar across both arms. Measurement of family experience, such as decisional conflict and bereavement, may also have been informative. Finally, dose measurement failed to incorporate case conference details, PCPC motivation and adherence to care plans, all of which may have affected EOL care. UC nursing homes can use more informal means other than case conferencing to share decisions with families (i.e. one-to-one conversations between nursing staff and families); such interactions where not captured in the dose measure but may have positively influenced EOL care and family satisfaction. It should also be noted that diagnosis-oriented medications are sometimes used with intent of symptom alleviation (e.g. corticosteroids for breathlessness from respiratory conditions) which may have led to underestimation of palliative pharmacological strategies.

## Conclusion

This study is one of the few RCTs of palliative care interventions in nursing homes worldwide, and appears to be the first to test efficacy of facilitated family case conferencing for people with advanced dementia. The study’s primary endpoint of quality of EOL care was under-powered and did not show evidence of effect. In spite of these limitations, a systematic approach to facilitating a palliative approach and skills enhancement drove improvements in care. Given the growing burden of dementia globally, these data will be formative in interventions aimed to improving palliative care in nursing homes in the future.

## Supporting information

S1 File Research protocol. (PDF)

S1 Table CONSORT (Consolidated Standards of Reporting Trials) checklist. (DOCX)
