# Supplementary material for: Effects of facilitated family case conferencing for advanced dementia: A cluster randomised clinical trial
Source: PLoS One. 2017 Aug 7;12(8):e0181020. doi: 10.1371/journal.pone.0181020 (PMC5546584; doi:10.1371/journal.pone.0181020)
Supplement: S1 File — (PDF) [file pone.0181020.s001.pdf]

RESEARCH PROTOCOL

1. FRONT PAGE

TITLE

Cluster randomised controlled trial of facilitated case conferencing versus usual care for improving end of life outcomes in aged care residents with advanced dementia and their families

VERSION: 1.1

DATE: 26th February 2013

LIST OF INVESTIGATORS AND PARTICIPATING INSTITUTIONS

Coordinating principal investigator:

Name	Meera Agar
Title	A/Prof
Qualifications	MBBS, FRACP, FAChPM, MPallCare
Positions held: employed, conjoint/adjunct/visiting	Director of the Dep't Palliative Care at Braeside Hospital, Wetherill Park; Research lead for South West Sydney Local Health District Palliative Care Service; Clinical trial director for the Ingham Institute of Applied Medical Research (South West Sydney); consultant physician in Palliative Medicine; Senior lecturer in Discipline of Palliative and Supportive Services, School of Medicine, Flinders University; Conjoint A/Prof at South West Sydney Clinical School, University of New South Wales; Conjoint A/Prof at School of Medicine University of Notra Dame; and Honorary Associate at the School of Public Health, School of Medicine, University of Sydney.
Full mailing address (including building	Dep't Palliative Care, Braeside Hospital, Locked Bag 82, Wetherill Park NSW 2164
Telephone	02 9616 8649
Fax	02 9616 8657
E-mail	Meera.agar@sswahs.nsw.gov.au

Co Investigators:

Name	Lynn Chenoweth
Title	Prof
Qualifications	RN, DipRec/leis (KCAE), BA (KCAE), GradCertHEd (UTS), MA (Hons) (Syd), MAdEd (UTS), PhD (Syd)
Positions held: employed,	Professor of Aged & Extended Care Nursing, University of Technology Sydney (UTS)
Full mailing address (including building	Faculty of Nursing, Midwifery & Health, Building 10, Level 7, UTS, 235-253 Jones St, Ultimo, NSW 2007
Telephone	0 2 9514 4857
Fax	02 9514 5049
E-mail	Lynn.chenoweth@uts.edu.au

Name	Geoffrey Mitchell
Title	Prof
Qualifications	MBBS, FRACP, PhD, FAChPM
Positions held: employed,	Professor of General Practice and Palliative Care, University of Queensland (UQ)
Full mailing address (including building	Building 12, UQ Ipswich Campus, Salisbury Rd,Ipswich, QLD 4305
Telephone	(0)7 3381 1363
Fax	(0)7 3381 1356
E-mail	g.mitchell@uq.edu.au

Name	Elizabeth Beattie
Title	Prof
Qualifications	BA, MA, PhD
Positions held: employed, conjoint/adjunct/visiting	Professor of Geropsychiatric and Dementia Nursing, Queensland University of Technology; Director, Dementia Collaborative Research Centre (DCRC) Carers and Consumers
Full mailing address (including building	N Block, Floor 6, School of Nursing Queensland University of Technology Victoria Park, Kelvin Grove, QLD 4059
Telephone	07-3138-3389
Fax	None
E-mail	elizabeth.beattie@qut.edu.au
Staff id (eg: z1234567)	NA

Name	Georgina Luscombe
Title	Dr
Qualifications	BSc, PhD
Positions held: employed, conjoint/adjunct/visiting	Adjunct Lecturer in Obstetrics, Gynaecology and Neonatology, Central Clinical School, University of Sydney
Full mailing address (including building	Central Clinical School, Room 408, Blackburn Bldg D06, University of Sydney, NSW 2006
Telephone	02 9351 3735
Fax	None
E-mail	Georgina.luscombe@sydney.edu.au

Name	Stephen Goodall
Title	Dr
Qualifications	BSc, MSc, PhD
Positions held: employed, conjoint/adjunct/visiting	Core Member, Centre for Health Economics Research & Evaluation (CHERE), UTS
Full mailing address (including building	CHERE, UTS, PO Box 123, Broadway NSW 2007
Telephone	02 9514 4752
Fax	02 9514 4730
E-mail	Stephen.goodall@uts.edu.au

Name	Dimity Pond

Title	Prof
Qualifications	BA (Hons), MBBS, FRACGP, Dip SSc, PhD
Positions held: employed, conjoint/adjunct/visiting	Professor in General Practice, University of Newcastle
Full mailing address (including building	School of Medicine and Public Health, Faculty of Health, University of Newcastle,
Telephone	(02) 4968 6720
Fax	(02) 4968 6727
E-mail	Dimity.pond@newcastle.edu.au
Staff id (eg: z1234567)	NA

Name	Jane Phillips
Title	Prof
Qualifications	RN BN PhD MRCNA
Positions held: employed, conjoint/adjunct/visiting	Professor Palliative Nursing, Cunningham Centre for Palliative Care Sydney, and The University of Notre Dame, Australia
Full mailing address (including building	Sacred Heart Hospice, 170 Darlinghurst St, Darlinghurst NSW 2010
Telephone	0411 100617
Fax	02 9357 7680
E-mail	Jane.phillips@nd.edu.au

Name	Tim Luckett
Title	Dr
Qualifications	BSc (Hons), PhD
Positions held: employed, conjoint/adjunct/visiting	Program Coordinator, Improving Palliative Care through Clinical Trials (ImPaCCT); Research Associate, South Western Sydney Clinical School, UNSW; Research Fellow, UTS
Full mailing address (including building	Faculty of Nursing, Midwifery & Health, Building 10, Level 7, UTS, 235-253 Jones St, Ultimo, NSW 2007
Telephone	02 95144861
Fax	02 9514 4474
E-mail	t.luckett@unsw.edu.au

Name	Patricia Davidson
Title	Prof
Qualifications	RN, BA, Med, PhD
Positions held: employed, conjoint/adjunct/visiting	Director of the Centre for Cardiovascular and Chronic Care, UTS
Full mailing address (including building	Faculty of Nursing, Midwifery & Health, Building 10, Level 7, UTS, 235-253 Jones St, Ultimo, NSW 2007
Telephone	02 95144822
Fax	02 9514 4835
E-mail	Patriciamary.davidson@uts.edu.au

Name	Meredith Gresham
Title	Ms
Qualifications	BAppSciOT, DipArts Mus, AMusA
Positions held: employed, conjoint/adjunct/visiting	Senior Dementia Consultant - Research & Design, HammondCare
Full mailing address (including building	Pallister House, 97-115 River Road, Greenwich NSW 2065
Telephone	02 84377300
Fax	None
E-mail	mgresham@hammond.com.au

Name	Victor Vickland
Title	Dr
Qualifications	PhD
Positions held: employed, conjoint/adjunct/visiting	Research Fellow
Full mailing address (including building	AGSM Building, Room 309, UNSW, Radwick, NSW 20152
Telephone	02 9385-2626
Fax	02 9385-2220
E-mail	Victor.vickland@unsw.edu.au

2. SYNOPSIS

Background

Dementia is a terminal disease. Care for people with advanced dementia requires a palliative approach that is targeted to the illness trajectory and tailored to the needs of each individual and his/her family. Currently, the quality of care in residential aged care (RAC) is compromised by lack of staff expertise and poor communication between staff, family and health professionals. Residents suffer unnecessary hospitalisations and aggressive treatments, while symptoms often go unmanaged. Facilitated case conferencing (FCC) is an approach that brings together RAC staff, health professionals and families to plan person-centred management based on best practice. FCC has improved outcomes in other palliative settings but evidence is lacking for RAC residents with advanced dementia.

Objectives

This phase III cluster RCT aims to: 1) compare the efficacy of FCC with Usual care in improving end of life (EOL) outcomes for residents with advanced dementia living in RAC; 2) provide insights into facility- and staff-related processes influencing the implementation and sustainability of FCC; and 3) evaluate the cost-effectiveness of FCC versus Usual care.

Study Plan

<u>Study setting</u>

Twenty RAC facilities (RACFs) in Sydney & Brisbane will be recruited.

Design and Methods

The proposed research will use a phase III parallel cluster RCT design, conducted in 6 stages over 3 years. A cluster RCT design was chosen to minimise contamination between arms when taking a systems-based approach to intervention. Activities for each stage will be as follows:

1. *Set-up*: Project staff recruitment; ethics approval; RACF eligibility screening; RACF level consents; RACF staff consents; recruitment & training of research staff (including inter-rater reliability).
2. *Pre-test:* Resident/family consent; baseline data collection at RACF, dyad & staff levels; identification, selection & appointment of PCPCs at Intervention RACFs.
3. *Intervention (training)* – The responsibility for FCC will be the role of Palliative Care Planning Coordinators" (PCPCs) - with continuous training for other RAC staff. This stage will include Palliative care planning coordinator (PCPC) baseline data collection and training by research staff; PCPC post-training evaluation of learning outcomes; ongoing support of case conferencing (CC) & PCPC training of other staff in the RACF:
4. *Delivery of FCC & follow-up* - Ongoing recruitment of any newly admitted residents who meet eligibility criteria until 27 months (to allow 6 months follow-up period for outcomes); organisation of CC at relevant trigger points (see below), 3-monthly collection, entering & checking of process & outcomes data at all sites; interviews with family two months following resident death.
5. *Final follow-up*: Final data collection from resident/family dyads & follow-up of residents with advanced dementia who were enrolled late in the project period; exit interviews with PCPCs & random samples of families, RAC staff & community health professionals involved in care at each RACF.
6. *Close-out*: Data checking, analysis & reporting; feedback to participants; analysis & reporting.

Randomisation & blinding: Randomisation will be 1:1 & occur after initial consent & baseline data at RACF, staff & resident/family levels. Research assistants will be blinded to the purpose of the study, & each will visit only RACFs in either the Intervention or Control arm. Project managers will need to be unblinded in order to liaise with PCPCs at Intervention RACFs; they will not collect process or outcome data after baseline.

Participants within each RACF: *Eligibility criteria for residents are:* a diagnosis of dementia & advanced disease as determined by Functional Assessment Staging Tool (FAST) in dementia ≥6a, stable for 1 month according to RACF staff; Australia–modified Karnofsky Performance Status (AKPS) of ≤50; availability of a person legally responsible to give consent on their behalf; informed consent from a family member or other who knows the resident well & will participate as the second member of the dyad. The FAST has been chosen because of its utility in the advanced stages of dementia; a FAST stage 7c & functional dependency (measured here by AKPS) is predictive of <6 months survival. *Eligibility for family/friend "informant"* (ideally the person legally responsible): visits the resident at least once a fortnight; knew the resident prior to their dementia diagnosis; is willing to be involved in decisions about the resident's care; 4) English proficiency sufficient to complete outcome measures (below). *RAC staff:* all permanent nursing/personal care staff. *Other health professionals* (e.g. GPs) involved in case conferencing for participating residents will also be eligible to participate.

Outcomes: *Primary:* Family-rated EOL outcomes will be measured using the End of Life in Dementia (EOLD) Scales.

The EOLD Scales will be used to measure symptom-related comfort during the last 7 days of life (CAD-EOLD), symptom management in the last 90 days of life (SM-EOLD) & family satisfaction with care during the last 90 days of life (SWC-EOLD). While the CAD-EOLD & SM-EOLD have been

validated for rating either by family or nurses, family ratings will be our primary outcome because family perceptions of EOL suffering & its management are important outcomes for palliative care & our intervention's staff focus may lead to response bias in nurse ratings. All scales will be rated 4 weeks following resident death.

Secondary:

a. Nurse-rated symptom-related comfort & symptom management will be measured using the CAD-EOLD & SM-EOLD Scales, & rated 4 weeks following residents' death by the nurse who has provided most care in the last 7 days & 90 days of life respectively.
b. Nurse-rated resident QOL will be measured second weekly using the Quality of Life in Late-stage Dementia (QUALID) Scale and EQ-5D-5L for economic evaluation purposes.
c. A palliative approach to care at the facility level will be measured using the following indices: rates of acute care episodes & length of stay (including ED presentations with admission & actual admission) for residents with advanced dementia; rates of inappropriate acute care episodes, as judged again by an expert review of 10% of admissions at each RACF. Documents reviewed will be nursing records, transfer letters, discharge summaries & medical records written on admission & discharge; rates of non-palliative interventions, defined as ventilation, resuscitation, nasogastric/PEG feeding & non-palliative pharmacological treatments; rates of inappropriate, non-palliative interventions, as judged by expert review of nursing records; rates of adverse events, identified via a review of nursing records & incident charts & coded as: falls with/without injury, skin tears, injuries during care & medication incidents; & number/type of complaints.
d. RACF person-centred approach to care will be measured by the 'Care & Activities, & Interpersonal Relationships & Interactions' domain of the Person-Centred Environment & Care Assessment Tool (PCECAT).

e. RAC staff's attitudes to, knowledge of & confidence in providing palliative/EOL care to residents with advanced dementia will be evaluated using the 35-item Palliative Care for Advanced Dementia (qPAD),

Analysis

Aim 1 – Efficacy: Primary & secondary analyses will be performed on an intention to treat (ITT) basis. Descriptive statistics will be used to compare characteristics at individual- & facility-levels in each group at baseline, accounting for the clustering effect in the former. Mixed or 'multilevel' modelling will be used to determine the effects of FCC & Usual care on primary & individual-level secondary outcomes (i.e. all secondary hypotheses except d). Mixed models allow adjustment for both individual (i.e. resident, family or staff) & cluster-level covariates as well as adjustment for the inherent correlation within clusters. Cluster level analyses will be implemented to determine the effect of the intervention on facility-level outcomes. Analyses will be weighted by cluster size as required. Results will be interpreted & generalised accordingly.

Aim 2 – Process & sustainability: Bayesian inference networks analysis (BINA) will be used to further model the influence of resident, family, staff, RACF & intervention variables on processes & outcomes. This analysis will overcome two limitations of traditional statistical analyses, namely: 1) inability to detect intervention effects due to smaller than expected effect size or heterogeneity in participants or facilities, & 2) relevance of results only to groups with certain 'average' characteristics that cannot be easily translated to each individual. BINA overcomes these problems by linking individuals' characteristics (e.g., severity of dementia) & events (e.g., change in clinical status) to estimate likelihoods of future improvement or deterioration. These models enable initial assumptions regarding efficacy to be modified & probabilities recalculated to accommodate new observations. Network analysis will also be used to model relationships between family & staff EOLD Scale ratings in each

arm to provide insight into potential direction & degree of response bias. BINA is widely applied in information technology (e.g., the Google search engine) but use in health research & dementia care is only just emerging & observational rather than evaluative data have been used. BINA will be carried out by Dr Victor Vickland, from the Dementia Collaborative Research Centre (DCRC) (UNSW).

Qualitative analysis of interview data will be supervised by CIs Chenoweth, Beattie & Phillips & will use standard coding & classifying techniques & N-Vivo software.

Aim 3 - Economic evaluation: The incremental benefit of FCC will be calculated in terms of QALYs gained. The cost of the intervention will be measured along with any cost-savings due to avoided healthcare utilisation. Results will be presented in terms of the incremental cost-effectiveness ratio (ICER). Many of these model parameters will not be powered for statistical significance. Therefore, mean estimates of resource utilisation will be used & confidence intervals will be generated by boot-strapping the data. Uncertainty will be explored using probabilistic sensitivity analysis.

3. RATIONALE / BACKGROUND

More than half (53%) of all Australians in RAC have a dementia diagnosis,[1] and most people with advanced dementia live in RAC.[2] Dementia is a terminal disease, and a palliative approach is fundamental to best practice care for people in the advanced stages.[2-5] According to the World Health Organisation, palliative care should "*improve the quality of life of individuals and their families … by means of early identification and impeccable assessment and treatment of pain and other problems, physical, psychosocial, cultural and spiritual needs".*[6] Unfortunately, delivery and quality of palliative care provision for people with advanced dementia in RAC is often suboptimal.[3,7-16]

What makes a dementia-specific palliative approach critical? Advanced dementia requires a palliative approach targeted to the specific needs of the illness trajectory as well as tailored to the needs of the individual and his/her family.[16] People with advanced dementia are usually unable to communicate their needs or participate in care decisions. Symptom assessment and management is in the context of severe cognitive impairment, symptom profiles are unique, and deterioration is often complicated by other life limiting conditions.[17] Pneumonia and eating problems are more common than in non-demented RAC residents and require focused management and planning.[18] Family members have defined a 'good death' for RAC residents with advanced dementia as requiring symptom management, clear decision-making, preparation, completion, and affirmation of the whole person.[18]

Current deficiencies in RAC dementia palliation: While there is substantial evidence to guide dementia-specific palliative care, many RAC staff and other health professionals lack awareness that a palliative approach is required, or else find it difficult to apply to dementia care.[11,13,19,20] Major barriers include deficiencies in expertise,[12,21] poor communication between services and between RAC staff and families, and inadequate planning and/or inconsistency in decision-making.[11,12] Symptoms such

as pain often go unrecognised and unmanaged.[12,15,22] Medical problems may be inappropriately managed with aggressive treatments (e.g. intravenous antibiotics/hydration, tube feeding),[13,20,23] compromising comfort for little or no survival benefit and reducing family satisfaction with EOL care.[14,24-26] If a palliative plan is not in place, staff may be unwilling or unable to manage problems in the facility, leading to transfer to hospital.[27] Hospitalisation may be frightening for residents, is associated with iatrogenic events, and may not be focused on QOL.[4,28] Hospital patients with dementia are more likely than non-demented to have a lengthy, terminal stay [29] and less likely to be treated palliatively and have caregivers involved in decisions.[30] These findings highlight the need to develop and evaluate models to support high quality palliative care for people with advanced dementia within RAC.[31] The policy frameworks relating to this imperative and relevant work undertaken by the current team are as follows.

Supporting RAC staff in delivering high quality dementia palliative care: The *National Framework for Action on Dementia 2006-2010 (NFAD)* [32] and *NSW Dementia Services Framework 2010-2015* [2] recognise the need to better train and resource RAC staff to deliver high quality palliative care, and both promote person-centred care (PCC). PCC strives to support 'personhood' and well-being of the person with dementia in all aspects of service provision.[33] Operationalising these directives in contemporary Australian aged care is extremely challenging due to variations in workforce profiles, high staff turnover, poor continuity of care and large numbers of unregulated and untrained workers employed to provide personal care.[34,35]

Learnings from evaluation of person-centred care (PCC): Dementia-specific PCC training for RAC staff in two cluster RCTs conducted by two investigators in this team, Prof Chenoweth and Dr Luscombe have shown capacity to directly impact resident symptoms and QOL.[36,37] Both trials supplemented training with mentoring and support to bring about a cultural change at each facility. In

the first trial, the "Caring for Aged Dementia Care Resident Study" (CADRES),[36] residents in PCC-trained residential aged care facilities (RACFs) were found to have decreased agitation and improved sleep, eating, drinking and QOL compared with Usual care. PCC was also cost-effective. The second trial, the "Person-Centred Environment and Care for residents with dementia" (PerCEN) study,[37] is currently in the follow-up stage with funding from the NHMRC and will evaluate effects of PCC and environment on residents' QOL and quality of care. CI Chenoweth and CI Beattie have also recently completed another PCC dementia project (EN-ABLE), providing evidence for the efficacy of facilitated staff training and support in promoting collaboration between staff and family in planning, implementing and monitoring quality of care.

The role of case conferencing (CC) in palliative care for RAC residents with advanced dementia: The *NSW Dementia Services Framework 2010-2015* identifies case conferencing as a key requirement of palliative care, and strong partnerships between RACFs, GPs and aged health care service to avoid unnecessary hospital admissions as a key requirement of RAC.[2] CC promotes PCC and effective communication and coordination between individual healthcare providers, clients and families. It has special potential for residents with advanced dementia because it: 1) facilitates the sharing of different perspectives regarding what each resident might have wanted and 2) provides a formal framework to discuss expected changes and decide management. Family communication with RAC staff and involvement in decision-making are important factors in determining their satisfaction with care.[14]

Evidence that case conferencing (CC) improves outcomes: Despite support for CC at both commonwealth and state levels, more evidence is needed to determine efficacy and cost-effectiveness in RAC residents with advanced dementia. Where evidence *is* available for CC is in the setting of community palliative care. Two RCTs carried out by other members of this team found CC

to result in better maintenance of physical and mental health measures in the month before death[38] and to decrease number of hospitalisations.[39] This work demonstrates the need to build staff knowledge and motivation to undertake CC and to offer detailed guidance on issues that require planning.[17,38-41] Uptake of CC is also facilitated by ensuring organisational and administrative support, with special attention to GP involvement.

Development & feasibility/pilot testing by the current team:

A CC model and toolkit for palliative care has been developed and successfully implemented in RAC by two investigators in this team: Prof Phillips and Prof Davidson.[42] This project resulted in 2-3 residents per week having CCs over a 6-month period and a 40% increase in GP involvement. The toolkit includes specific, practical guidance and a template for initiating, setting-up, conducting and monitoring CCs and resulting care plans.[43] Associate Professor Agar (lead investigator) has since adapted this toolkit for residents with advanced dementia and piloted it in six large (200+ bed) RACFs in metropolitan and rural NSW.

This pilot has tested the key components to be utilised in the proposed intervention and has demonstrated:

1) acceptability of the advanced dementia-specific CC toolkit, the list of core skills for the coordinator role and associated training materials;

2) feasibility and appropriateness of dementia-specific "trigger points" based on resident characteristics and/or clinical events after which a CC is recommended;

3) acceptability by RAC staff of a palliative care framework that enables them to map the trajectory of residents in specific domains of disease progression (functioning, swallowing, weight loss, continence,

level of consciousness, communication, and factors related to comorbid illness) and identify the issues a related CC should address; and

4) feasibility of collecting descriptive data and outcome measures in the proposed evaluative study.

Baseline data collected in the pilot confirms previous findings that, without facilitation, CC does not occur;[44] over a 6-month observation period, less than 20 CCs took place, despite executive support. Staff communication with families was limited to exchange of information rather than shared decision-making. The pilot will be completed in June 2011, with thus far 60 residents meeting the triggers for CC and 10 facilitated CCs successfully undertaken utilising the framework in a 2 month period.

Approach in the proposed research: This application will bring together the team's experiences in palliative care CC (Agar/Mitchell/Phillips) and PCC-related RAC cultural change (Chenoweth/Beattie/Luscombe) to implement and evaluate a model of sustainable FCC for residents with advanced dementia in RAC. Training and materials in CC and palliative care described above [40,43] will be implemented together with an adapted version of the PCC training and support package evaluated by Prof Chenoweth.[36,37] Our pilot work highlights that a "bottom up" approach utilising purely transient and less skilled staff is not effective. A more practical approach is to include responsibility for CC within the core, paid duties of specialised staff trained for the role – "Palliative Care Planning Coordinators" (PCPCs) - with continuous training for other RAC staff. The pilot work referred to above found use of a specially trained coordinator increased referrals to specialist palliative care by 90%.[42]

FCC for residents with advanced dementia in RAC is a complex intervention, as defined by the UK Medical Research Council (MRC).[45] Complex interventions require tailored implementation to local contexts and monitoring of processes to assess fidelity of implementation, clarify causal mechanisms, and identify influential contextual factors to inform ongoing development and evaluation. As noted

above, a *development phase* has been completed over a number of projects and a *feasibility and piloting ph*ase will end in June 2011. The current project is the *evaluation phase* aimed at determining efficacy, understanding change process within the RACFs and evaluating cost effectiveness. This will inform dissemination, surveillance and monitoring, and longer-term follow-up in a future *implementation phase* for which further funding will be requested. The intervention will be implemented using the UK's National Institute for Health and Clinical Excellence guidance on behaviour change, which advocates partnerships with those involved to ensure interventions are appropriate to local need.[46]

4. AIMS / OBJECTIVES / HYPOTHESES

Aims

This phase III cluster RCT aims to: 1) compare the efficacy of FCC with Usual care in improving end of life (EOL) outcomes for residents with advanced dementia living in RAC; 2) provide insights into facility- and staff-related processes influencing the implementation and sustainability of FCC; and 3) evaluate the cost-effectiveness of FCC versus Usual care.

Hypotheses

Primary hypothesis (Aim 1):

Compared with Usual care, FCC for residents with advanced dementia will achieve better family-rated EOL outcomes as defined by: a) better symptom-related comfort in the last 7 days of life; b) more

effective symptom management over the last 90 days of life; c) greater family satisfaction with care over the last 90 days of the resident's life.

<u>Hypothesis 2 (Aim 2):</u>

Compared with Usual care, FCC for residents with advanced dementia will achieve:

a) better nurse-rated symptom-related comfort over the last 7 days of life and symptom management over the last 90 days of life;

b) higher scores on resident quality of life (QOL) at last measurement prior to resident death;

c) a palliative approach to medical care of residents with advanced dementia, as evidenced by reduced acute care episodes, non-palliative interventions and adverse incidents at the facility level;

d) a person-centred approach by RACF staff to all aspects of care;

e) improvements in RAC staff attitudes to, knowledge of and confidence in providing palliative care to residents with advanced dementia and dementia care more generally.

f) improvements in family QOL.

<u>Hypothesis 3 (Aim 3):</u> Compared with Usual care, FCC for residents with advanced dementia will EITHER result in an overall reduction in health care costs with no loss to resident health OR be cost-effective in terms of health gained at acceptable additional cost.

5. PARTICIPATING SITES

Twenty RACFs in Sydney and Brisbane will be recruited (10 per arm). RACFs likely to meet the eligibility criteria will be identified from lists and approached in random order.: 1) ≥100 beds, 2) ≥50% residents with dementia, 3) identified as high care. RACFs meeting these criteria will be approached

by letter and phone to canvass interest. Informed consent for facility participation will then be obtained from facility managers.

6. RESEARCH PLAN / STUDY DESIGN

6.1 Study design

The proposed research will use a phase III parallel cluster RCT design, conducted in 6 stages over 3 years. A cluster RCT design was chosen to minimise contamination between arms when taking a systems-based approach to intervention.

<u>Activities for each stage will be as follows:</u>

1. *Set-up*: Project staff recruitment; application for ethics approval; identification of eligible RACFs and randomised approach; RACF level consents; RACF staff consents; recruitment and training of research staff (including inter-rater reliability).

2. *Pre-test:* Collection of resident/family consent; baseline data collection at RACF, dyad and staff levels; identification, selection and appointment of PCPCs at each Intervention RACFs.

3. *Intervention (training)* - PCPC baseline data; training; PCPC post-training evaluation of learning outcomes; ongoing support of CC and PCPC training of other staff;

4. *Delivery of FCC and follow-up* - Ongoing recruitment of any newly admitted residents who meet eligibility criteria until 27 months (to allow 6 months follow-up period for outcomes); organisation of CC at relevant trigger points (see below), 3-monthly collection, entering and checking of process and outcomes data at all sites; interviews with family two months following resident death.

5. *Final follow-up*: Final data collection from resident/family dyads and follow-up of residents with advanced dementia who were enrolled late in the project period; exit interviews with PCPCs and random samples of families, RAC staff and community health professionals involved in care at each RACF.

6. *Close-out*: Data checking, analysis and reporting; feedback to participants; analysis and reporting.

Conditions: The Intervention and Usual care to be compared in the proposed study will be as follows.

INTERVENTION:

We will use a train-the-trainer model, whereby a Palliative Care Planning Coordinator (PCPC) is trained and then supported to train other nursing and personal care staff at each RACF.

Palliative Care Planning Coordinators (PCPCs): RACFs randomised to the Intervention arm will be invited to discuss allocation of PCPC responsibility best-suited to local conditions. The PCPC will be the team leader in each RACF responsible for implementing the CC model as per the protocol and providing ongoing education and mentoring to other RACF staff (see below). Our pilot experience suggests the role will best suit a permanent, full-time, senior nurse. (S)he will require communication skills and clinical expertise appropriate to dementia and palliative care. PCPCs will be expected to commit 0.5FTE to the role, paid at their usual rate. Selection will be via interview and, once appointed, relevant duties will be added to the staff member's job description. PCPCs will attend one week full time equivalent of training at a central location. To assess learning outcomes, PCPCs will complete pre- and post training administrations of the Palliative Care for Advanced Dementia (qPAD), a 35-item tool that assesses health professionals' knowledge of the care needs of people with advanced dementia and attitudes towards providing related care.

PCPC training in advanced dementia-specific FCC and person-centred, palliative/EOL care: PCPCs will learn to *identify 'triggers' that warrant initiation of a FCC,* including: admission to RACF; return to RACF following discharge from acute care or Emergency Department (ED) presentation; increase in falls; change in clinical status; new/worsening symptoms; poor appetite; poor skin integrity; at the annual management plan review in conjunction with the Aged Care Funding Instrument [ACFI];[47] receipt of a complaint; family disagreement about care; family distress.

Where a trigger for FCC has been identified, the point of disease progression will be mapped onto relevant domains to indicate whether survival time will likely be measured in months, weeks or days. The "map" for each resident will highlight issues for discussion at CC, including preemptive planning for predicted deterioration. The PCPC will receive training in organising CCs and receive *specific guidance on when and how to initiate advance care planning regarding do-not-resuscitate (DNR) and do-not-hospitalise (DNH) orders and artificial nutrition/hydration*.[48]

Training in facilitating general practitioner involvement: Strategies will include using a direct telephone approach with an offer of a follow-up visit to discuss, assistance with obtaining remuneration for time spent, and ensuring that FCCs are as time-efficient as possible. Wherever possible, GPs will be given advance notice of FCCs and asked to book them in as appointments. FCCs will be arranged at times to suit GPs (e.g., lunch times), and GPs will be given the opportunity to join by teleconference only for those CC contents most relevant to them (e.g., symptom management). These approaches have led to impressive GP attendance rates in a previous RCT by the team.[38]

Palliative environment: Finally, training will assist RACF staff to provide a "palliative" space (an area which provides the resident and family a quiet, homelike private environment 24 hours per day.[49]

PCC training will make use of experiential and adult learning approaches, guidance on how family members can be involved as much as possible in decision-making about care planning, implementation and monitoring, and support reconceptualisation from a "service focus" to person-centred focus.[50,51]

Train-the-trainer training will provide PCPCs with the skills and resources to deliver one-day training and mentoring in advanced dementia-specific CC and PCC palliative care to other nurses and care staff. Ongoing support in the Intervention arm will be provided following initial training and for the rest of the study period. The team will liaise monthly with each PCPC to discuss progress and difficulties and plan support of staff training and CCs where monitoring data (see below) indicate this is required.

<u>USUAL CARE</u>:

There will be no additional education, training or support provided to Control RACFs but there will also be no restriction on service-provider education and training where this is current practice. The level, duration and type of such initiatives will be documented.

Randomisation and blinding: Block randomisation will occur after initial consent & baseline data at RACF level using two stratification factors (dementia specific unit or not; part of organisation or stand alone facility) using a computer generated allocation sequence. The statistician allocating sites will be blind to their identity. Research assistants will be blinded to the purpose of the study, & each will visit only RACFs in either the Intervention or Control arm. Project managers will need to be unblinded in

order to liaise with PCPCs at Intervention RACFs; they will not collect process or outcome data after baseline.

6.2 Data sources/Collection

Baseline data: Variables that have potential to influence outcomes will be tracked to inform further development and implementation.[45] Descriptive data will be collected as follows using adapted proformas from the CADRES [36] and PerCEN [37] studies.

Facility:

Number of beds; private versus not-for-profit status; organisational affiliation; dementia-specific status; accommodation and amenities; proportion of residents with dementia; resident to staff ratio; management structure and staff profile; involvement of GPs and other community services; staff turnover; use of agency staff; availability of therapy and recreation programs; proportion of residents requiring complex care; care planning meeting(s) involving family (‘CC’ or otherwise) for residents with advanced dementia in the past year, Person-Centred Environment and Care Assessment Tool (PCECAT) [52] score.

Staff:

Self-reported age, gender, qualifications, position, time in position, time in RAC, dementia experience and previous staff training.

Resident:

Age; gender; previous occupation; years of education; time since dementia diagnosis; length of stay in RAC; family members; number of visitors and frequency of visits; most recent Aged Care Funding Instrument (ACFI) report [47] (administered by trained staff and includes assessment of comorbidities, activities of daily living, cognitive functioning, behavioural and psychological symptoms, medication use, complex care needs and continence; pressure ulcers; food and fluid intake; body mass index (BMI); PCOC phase;[53] and cognitive and communicative functioning as assessed by the Bedford Alzheimer Nursing Severity (BANS) Scale,[54] a measure specific to low functioning and used in a current large, US cohort study of advanced dementia - CASCADE.[55]

Family member:

Self-reported relationship to resident, age, gender, education, occupation, dependents, frequency/duration of visits, person responsible status and prior involvement in decision-making.

<u>Process and sustainability data:</u>

This will be collected to determine ‘dose’ and ‘duration’ of the study intervention and describe facilitative or obstructive mechanisms that can be used to inform future implementation.[45] Process data will be collected at Intervention arm RACFs using an adapted version of the PerCEN study process data schedule. Where relevant, data will inform the need for trouble-shooting. Data will include: PCPC and other staff attendance at training; PCPC and other staff learning outcomes (pre-/post-training and 6-month follow-up qPAD); CC numbers, triggers, duration and attendance; care plan content and adherence; quality of documentation. Data will be collected via reviews of nursing and CC records, interviews with PCPCs and observation of CCs. Assessment of adherence will be undertaken by a blinded, expert panel (including a palliative care physician, GP and RN) who will review documentation for a random 10% of residents at each RACF. The first 15 CCs observed by each research assistant will be audio-taped and independently analysed to validate observation-based

coding. Sustainability data will be collected via exit interviews with all PCPCs and a random sample of up to 30 families, RAC staff and community health professionals caring for enrolled residents in each arm (precise numbers will be determined by saturation).[56] Interviews will be semi-structured and will explore perceptions of PCC and palliative care for advanced dementia and the importance of multi-disciplinary and family involvement in care planning. In the Intervention arm, interviews will also concern perceptions of the intervention's usefulness and facilitators/barriers to its implementation. PCPCs will also be asked to give examples of how they have used the training/materials to improve the care of individual residents as well as to describe their approach to a range of hypothetical scenarios

<u>Outcome data:</u> Intervention and Control arms will be compared on the following outcomes:

Primary (Hypothesis 1): <u>Family-rated EOL outcomes</u> will be measured using the End of Life in Dementia (EOLD) Scales,[57] developed by Al Volicer. These scales have established internal consistency (Cronbach's alpha=0.68–0.83) and convergent validity in the RACF setting [58] and were recently rated the best measures for evaluating palliative care in RAC.[59] EOLD data from the CASCADE study will enable comparison with US residents.[55] The EOLD Scales will be used to measure: a) symptom-related comfort during the last 7 days of life (Comfort Assessment in Dying with Dementia; CAD–EOLD). The CAD-EOLD assesses physical distress, dying symptoms, emotional distress and wellbeing; b) symptom management in the last 90 days of life (Symptom Management at the End-of-Life in Dementia; SM–EOLD); and c) family satisfaction with care during the last 90 days of life (Satisfaction with Care at the End of life in Dementia; SWC–EOLD). While the CAD-EOLD and SM-EOLD have been validated for rating either by family or nurses, family ratings will be our primary outcome because family perceptions of EOL suffering and its management are important outcomes for palliative care [70] and our intervention's staff focus may lead to response bias in nurse ratings. All scales will be rated 4 weeks following resident death.

Secondary (Hypothesis 2):

a. Nurse-rated symptom-related comfort and symptom management will be measured using the CAD-EOLD and SM-EOLD Scales, and rated 4 weeks following residents' death by the nurse who has provided most care in the last 7 days and 90 days of life respectively. Nurse-ratings have been included because they may offer an alternative and more 24-hour perspective [60] and comparison with family ratings will allow exploration of potential bias from both kinds of respondents.

b. Nurse-rated resident QOL will be measured second weekly using the Quality of Life in Late-stage Dementia (QUALID) Scale.[61-63] This 11-item instrument is the only advanced dementia-specific QOL measure.[64] It has shown convergent validity, internal consistency (Cronbach's alpha=0.77) and test-retest (ICC=0.81) and inter-rater (k=0.83) reliability.[61] QUALID data from CADRES [36] and CASCADE [55] will enable comparison with large Australian and US samples. For the purposes of economic evaluation, QOL will also be assessed by proxy using the EQ-5D-5L,[65] a new version of the world's most widely used multi-attribute utility instrument EQ-5D [65] that has been revised to include a larger number of severity levels among its response options (therefore giving greater sensitivity). The EQ-5D has been widely used in dementia studies.[66]

c. A palliative approach to care at the facility level will be measured using the following indices: rates of acute care episodes and length of stay (including ED presentations with admission and actual admission) for residents with advanced dementia; rates of inappropriate acute care episodes, as judged again by an expert review of 10% of admissions at each RACF. Documents reviewed will be nursing records, transfer letters, discharge summaries and medical records written on admission and discharge; rates of non-palliative interventions, defined as ventilation, resuscitation, nasogastric/PEG feeding and non-palliative pharmacological treatments; rates of inappropriate, non-palliative interventions, as judged by expert review of nursing records; rates of

adverse events, identified via a review of nursing records and incident charts and coded as: falls with/without injury, skin tears, injuries during care and medication incidents; and number/type of complaints from families regarding the quality of care.

d. RACF person-centred approach to care will be measured by the 'Care and Activities, and Interpersonal Relationships and Interactions' domain of the Person-Centred Environment and Care Assessment Tool (PCECAT).[52] This tool, developed by CIB Chenoweth's team, requires a trained research assistant to assign a score between 0 (not at all) and 3 (all of the time) to the frequency of 18 qualities of care as evidenced by observation, resident/family reports and documentation.

e. RAC staff's attitudes to, knowledge of and confidence in providing palliative/EOL care to residents with advanced dementia will be evaluated using the Palliative Care for Advanced Dementia (qPAD).[67] This 35-item scale has demonstrated satisfactory factor structure and internal consistency (Cronbach's alpha 0.58 – 0.90). The qPAD will be administered before and after training to PCPCs by research staff and to other RAC staff by the PCPC delivering training. Responses from RAC staff will be anonymous to reduce response bias.

Secondary (Hypothesis 3): The economic evaluation will take a cost-utility approach, in which *health benefit* will be estimated in terms of quality adjusted life years (QALYs) gained. This will capture expected intervention effects on morbidity within the context of any unexpected change in survival. Resident QOL will be estimated using nurse-ratings on the EQ-5D-5L introduced above, for which preference weights have been valued by a representative sample of the general population. *Healthcare costs* from a societal perspective will include: training costs (materials, trainer's time and opportunity cost of trainee time); CC costs (session time, travel time and distance travelled by attendees); routine healthcare costs (use of GPs, specialists and pharmaceuticals).

6.3 Population/Sample size

Inclusion criteria

Residents

A diagnosis of dementia and advanced disease as determined by Functional Assessment Staging Tool (FAST)[68] in dementia ≥6a, stable for 1 month according to RACF staff; Australia–modified Karnofsky Performance Status (AKPS) [69] of ≤50; availability of a person legally responsible to give consent on their behalf; informed consent from a family member or other who knows the resident well and will participate as the second member of the dyad. The FAST has been chosen because of its utility in the advanced stages of dementia; a FAST stage 7c and functional dependency (measured here by AKPS) is predictive of <6 months survival.[70]

Family members (ideally the person legally responsible)

Visits the resident at least once a fortnight; knew the resident prior to their dementia diagnosis; is willing to be involved in decisions about the resident's care; English proficiency sufficient to complete outcome measures.

RAC staff

Permanent nursing, assistant in nursing and careworkers employed at a participating facility.

Health professionals

Health professionals (e.g. GPs) involved in case conferencing for participating residents.

Sample size

In the absence of established minimal clinically important differences on the EOLD Scales, we will follow a common rule-of-thumb and assume 0.5 standard deviation (SD).[71] Sample size has been based on the EOLD scale with the highest intra-cluster correlation (ICC) (i.e., requiring the greatest number of clusters). To detect a change in CAD-EOLD of half a standard deviation (a change in score of at least 3), with a two-sided 5% significance level, power of 80% and an intracluster correlation coefficient of 0.05 (estimated from unpublished data sourced from Dutch RAC facilities), a sample size of 8 clusters per group (16 in total), with 15 residents per cluster, was necessary. Given an anticipated dropout rate (at the resident level) of 10%, a recruitment sample of 272 residents (17 per site) would be needed for a final sample of 240 residents. An interim check conducted on blinded data as of 6 months data collection will be performed to assess whether any of these assumptions requires adjustment and a re-estimation of sample size is necessary. The feasiblity of accruing 17 residents with advanced dementia per RACF with a life expectancy less than the study period (<18 months) is based on: 1) a review of death data at Hammond Care RACFs, and 2) research showing that 40% of people admitted to RAC with advanced dementia die within 1 year and our eligibility criteria predict further decreases in life expectancy.[70,72]

6.4 Expected duration of study and start times

This 3-year study is expected to begin in September 2012 and be completed by June 2015.

6.5 Statistical analyses

Aim 1 – Efficacy: Primary and secondary analyses will be performed on an intention to treat (ITT) basis. Descriptive statistics will be used to compare characteristics at individual- and facility-levels in each group at baseline, accounting for the clustering effect in the former. Mixed or 'multilevel' modelling will be used to determine the effects of FCC and Usual care on primary and individual-level secondary outcomes (i.e. all secondary hypotheses except d). Mixed models allow adjustment for both individual (i.e. resident, family or staff) and cluster-level covariates as well as adjustment for the inherent correlation within clusters. Cluster level analyses will be implemented to determine the effect of the intervention on facility-level outcomes (i.e. hypothesis 2d). Analyses will be weighted by cluster size as required. Results will be interpreted and generalised accordingly.

Aim 2 – Process and sustainability: Bayesian inference networks analysis (BINA)[73,74] will be used to further model the influence of resident, family, staff, RACF and intervention variables on processes and outcomes. This analysis will overcome two limitations of traditional statistical analyses, namely: 1) inability to detect intervention effects due to smaller than expected effect size or heterogeneity in participants or facilities, and 2) relevance of results only to groups with certain 'average' characteristics that cannot be easily translated to each individual. BINA overcomes these problems by linking individuals' characteristics (e.g., severity of dementia) and events (e.g., change in clinical status) to estimate likelihoods of future improvement or deterioration. These models enable initial assumptions regarding efficacy to be modified and probabilities recalculated to accommodate new observations. Network analysis will also be used to model relationships between family and staff EOLD Scale ratings in each arm to provide insight into potential direction and degree of response bias. BINA is widely applied in information technology (e.g., the *Google* search engine) but use in health research and dementia care is only just emerging and observational rather than evaluative data

have been used.[75,76] BINA will be carried out by Dr Victor Vickland, from the Dementia Collaborative Research Centre (DCRC) (UNSW). Dr Vickland is CIA on an NHMRC-funded project using BINA to develop computer-assisted clinical guidelines of anxiety and depression management. *Qualitative analysis* of interview data will be supervised by CIs Chenoweth, Beattie and Phillips and will use standard coding and classifying techniques and N-Vivo software.[56]

Aim 3 - Economic evaluation: The incremental benefit of FCC will be calculated in terms of QALYs gained. The cost of the intervention will be measured along with any cost-savings due to avoided healthcare utilisation. Results will be presented in terms of the incremental cost-effectiveness ratio (ICER). Many of these model parameters will not be powered for statistical significance. Therefore, mean estimates of resource utilisation will be used and confidence intervals will be generated by boot-strapping the data. Uncertainty will be explored using probabilistic sensitivity analysis.

7. ETHICAL CONSIDERATIONS

7.1 Recruitment and selection of participants

Aged care facilities will be selected for approach from comprehensive lists of all facilities in each geographical area by means of a randomisation schedule intended to minimize selection bias. Facility managers will be approached via letter and telephone by the research team to canvass their initial interest. Managers expressing initial interest will be sent an information and consent form, followed by a follow-up phone call.

Facility managers and staff will be asked to identify residents who: 1) have dementia, in the advanced stages (with or without a confirmed diagnosis); 2) have a family member or friend who visits on a

regular basis; and 3) have a person responsible to give consent to participate on their behalf, whether this be the regular visitor or another person. The person responsible and the regular visitor will be given an information and consent form by facility staff either in person or by post and invited to contact the research team by telephone if they would like more information. A cover letter from the facility introducing the study will be included in post-outs, which may also be followed up by telephone. Upon contact, research staff will go through the procedure of informed consent, making it clear that participation is voluntary and will not affect relationships with the facility staff or researchers. Persons legally responsible who wish to will give informed consent on behalf of the resident with advanced dementia while regular visitors will be invited to give informed consent on their own behalf. Note that, in many cases, we expect the person responsible and regular visitor to be one and the same person so that they will give two consents – one on behalf of the resident with advanced dementia and one on their own behalf.

Facility staff with care responsibilities (i.e. nurses and assistants in nursing) will also be eligible to participate. Staff will be told about the study verbally at staff meetings, provided with information and consent forms and invited to contact the research team if they have further questions or would like to participate.

In facilities randomized to receive the intervention, a key participating staff member will be a palliative care planning coordinator (PCPC) who will be employed 0.4FTE to coordinate case conferencing at their facility. Candidates for the PCPC position will, wherever possible, be identified by managers from existing senior nursing staff using standardised criterion. Potential candidates will be given a duty statement by the manager and invited to contact the research team if they would like more information.

Finally, other health professionals who take part in case conferences for participating residents will also be eligible to take part. Their decision whether or not to participate in the evaluation will be quite independent of their decision to contribute to case conferences. The PCPC, who will have liaised with these health professionals to invite them to case conferences, will provide them with information and consent forms and invite them to contact the research team if they are willing to participate.

7.2 Informed consent

Informed consent will be collected from persons responsible (on behalf of residents), family members, RAC staff and health professionals. Persons responsible will receive and sign a study-specific patient information sheet and consent form (PIS/CF). A waiver will be sought from the Human Research Ethics Committee (HREC) for informed consent relating to summary data at the facility level used to monitor the quality of care (e.g. number of admissions, accidents, complaints).

7.3 Confidentiality and Privacy

Participant names will be replaced by ID numbers at the sites soon after data has been collected. A file linking ID numbers with names will be stored in a password protected folder at the study sites. Consent forms will be kept in a separate locked filing cabinet also at the study sites. The electronic file linking ID numbers with names will not leave the study sites and will be destroyed at the end of the project period once the need to locate data for removal in the case of withdrawal has passed.

All reports will be at the summary level and will not refer to individuals in any way that would enable their identification.

7.4 Data storage and Record retention

All data will be stored on password protected computers at the University of Technology Sydney's Faculty of Midwifery and Health and/or uploaded onto the CareSearch (www.caresearch.com.au) website research data management system which is password protected and encrypted, specifically developed for multicentre clinical trials. Access will vary according to the roles of individuals involved in the project. A/Prof Meera Agar, Dr Tim Luckett, Dr Georgina Luscombe, Dr Victor Vickland, a health economics research fellow, research assistants and a data manager will have access to all data either because they have collected and uploaded it, to monitor data quality or to prepare the data for analysis. Other members of the research team will have access only to summary data to contribute to interpretation.

The files linking ID numbers with personal details will be destroyed at the end of the project period after the need to locate data for removal in the event of participant withdrawal has passed. Other data will be maintained for 7 years after study completion. This meets national requirements for record retention of research materials. Once the waiting period is complete, we will erase all electronic files (including those on the CareSearch website) and shred any paper copies.

8. OUTCOMES AND SIGNIFICANCE

Dementia is now Australia's third leading cause of death and is increasing exponentially.[77] In RAC, dementia is the greatest single contributor to the cost of care.[78] The total cost of dementia in 2002 was $6.6 billion – over $40,000 per annum per person with dementia; this is expected to grow to 3.3% of GDP by 2050.[79] Hospital stays for people with dementia may be twice as long and ED costs 2.5 times

greater than for non-demented elderly.[80] Research suggests that many hospitalisations occur for non-palliative, unnecessary reasons.[20] The draft Productivity Commission report *Caring for Older Australians* [81] also suggests an increase in transfers of RAC residents with dementia to hospital for pain management and EOL care due to insufficient staff expertise and limited GP involvement (p.260). The Commission calls for more palliative care in RAC and the education and resources to support this. By addressing this recommendation, our intervention will reduce healthcare burden while improving care quality, resident wellbeing and family satisfaction. Although CC in RAC has been widely promoted, uptake is fragmented and the impact on outcomes poorly understood. Our intervention is novel in that it is targeted to the dementia trajectory, tailored to individual RACF, resident and family needs, and is strongly aligned to RAC business processes. These qualities will deliver an important contribution to the Government's aged care reform agenda.

9. TIMELINES / MILESTONES

The timeline for the 6 project stages is summarised in Table 1.

Table 1. Timing of the 6 project stages

**Stage**	**Project period (in months)** 0-6	**7-12**	**13-18**	**19-24**	**25-30**	**31-36**
1. Set-up	■					
2. Pre-test	■	■				
3. Intervention (training)		■				
4. FCC and follow-up			■	■	■	
5. Final follow-up						■
6. Close-out						■

10. PUBLICATION POLICY

Any Intellectual Property arising from this study shall be jointly owned by the collaborating research team. There have been no limits placed on publication of results.

11. REFERENCES

1. Australian Institute of Health and Welfare. *Residential Aged Care in Australia 2007-2008.* Canberra: AIHW;2009.

2. NSW Dementia Policy Team. *The NSW Dementia Services Framework 2010-2015.* Sydney: NSW Department of Health;2010.
3. Alzheimers Australia. *Palliative care and dementia.* Brisbane: Alzheimers Australia;2006.
4. Hines S, et al. *Effectiveness and appropriateness of a palliative approach to care for people with advanced dementia: A systematic review.* Brisbane: Dementia Collaborative Research Centre for carers and consumers, QUT;2009.
5. Sampson EL. Palliative care for people with dementia. *Br Med Bull.* December 2010;96 (1):159-174.
6. Sepulveda CM, *et al.* Palliative Care: The World Health Organisation's Global Perspective. *Journal of Pain and Symptom Management.* 2002;24:91-96.
7. Kristjanson L, *et al.* End of LIfe Challenges in Residential Aged Care Facilities: A Case for a Palliative Approach to Care. *Int J Palliat Nurs.* 2005;11(3):127-129.
8. Commonwealth of Australia. *Guidelines for a palliative approach in residential aged care.* Canberra: National Health and Medical Research Council;2006.
9. Phillips J, *et al.* Residential aged care: the last frontier for palliative care. *J Adv Nurs.* Aug 2006;55(4):416-424.
10. Teno JM. Now is the time to embrace nursing homes as a place of care for dying persons. *J Palliat Med.* Apr 2003;6(2):293-296.
11. Birch D, Draper J. A critical literature review exploring the challenges of delivering effective palliative care to older people with dementia. *J Clin Nurs.* May 2008;17(9):1144-1163.
12. Chang E, *et al.* Challenges for Professional Care of Advanced Dementia. *Int J Nurs Pract.* 2009;15:41-47.
13. Di Giulio P, *et al.* Dying with Advanced Dementia in Long-Term Care Geriatric Institutions: A Retrospective Study. *J Palliat Med.* 2008;11(7):1023-1028.
14. Engel SE, *et al.* Satisfaction with End-of-Life Care for Nursing Home Residents with Advanced Dementia. *J Am Geriatr Soc.* 2006;54(10):1567-1572.
15. Bayer A. Death with dementia--the need for better care. *Age Ageing.* Mar 2006;35(2):101-102.
16. ACH Group. *Planning for Palliative Dementia Care: Resource Guide.* Adelaide: ACH Group;2009.
17. Mitchell SL, *et al.* The clinical course of advanced dementia. *New England Journal of Medicine.* Oct 15 2009;361(16):1529-1538.
18. Bosek MS, *et al.* Promoting a good death for persons with dementia in nursing facilities: family caregivers' perspectives. *JONA'S healthcare law, ethics and regulation.* Jun 2003;5 (2):34-41.
19. Hertogh CMPM. Advanced Care Planning and the Relevance of a Palliative Care Approach in Dementia. *Age and Ageing.* 2006;35:553-555.
20. Mitchell SL, *et al.* Dying with advanced dementia in the nursing home. *Arch Intern Med.* Feb 9 2004;164(3):321-326.
21. Rurup ML, *et al.* Attitudes of physicians, nurses and relatives towards end-of-life decisions concerning nursing home patients with dementia. *Patient Educ Couns.* Jun 2006;61(3):372-380.
22. McAuliffe L, *et al.* Pain Assessment in Older People with Dementia:Literature Review. *J Adv Nurs.* 2009;65(1):2-10.
23. Sampson EL, *et al.* Differences in care received by patients with and without dementia who died during acute hospital admission: a retrospective case note study. *Age Ageing.* Mar 2006;35(2):187-189.

24. Givens JL, *et al.* Survival and comfort after treatment of pneumonia in advanced dementia. *Arch Intern Med.* Jul 12 2010;170(13):1102-1107.
25. Meier DE, *et al.* High short-term mortality in hospitalized patients with advanced dementia: lack of benefit of tube feeding. *Arch Intern Med.* Feb 26 2001;161(4):594-599.
26. Sorrell JM. Use of feeding tubes in patients with advanced dementia: Are we doing harm? *Journal of Psychosocial Nursing and Mental Health Services.* May 2010;48 (5):15-18.
27. Mitchell SL, *et al.* Decisions to forgo hospitalization in advanced dementia: a nationwide study. *J Am Geriatr Soc.* Mar 2007;55(3):432-438.
28. Andrews J, Christie J. Emergency care for people with dementia. *Emerg Nurse.* 2009;17(5):12.
29. Guijarro R, *et al.* Impact of dementia on hospitalization. *Neuroepidemiology.* Aug 2010;35(2):101-108.
30. Afzal N, *et al.* Quality of end-of-life care for dementia patients during acute hospital admission: a retrospective study in Ireland. *Gen Hosp Psychiatry.* Mar-Apr 2010;32(2):141-146.
31. Sampson EL, *et al.* A systematic review of the scientific evidence for the efficacy of a palliative care approach in advanced dementia. *Int Psychogeriatr.* Mar 2005;17(1):31-40.
32. Australian Health Ministers' Conference (AHMC). *National Framework for Action on Dementia (NFAD).* North Sydney: NSW Department of Health on behalf of AHMC;2006.
33. Kitwood T. Culture of care: tradition and change. In: Kitwood T, Benson S, eds. *The New Culture of Dementia Care*. London: Hawker Publications; 1999
34. Chenoweth L, Kilstoff K. Organizational and structural reform in aged care organizations: empowerment towards a change process. *J Nurs Manag.* Jul 2002;10(4):235-244.
35. Department of Health and Ageing. *Final report: Research study into the educational, training and support needs of general practitioners in palliative care.* Canberra, ACT2003.
36. Chenoweth L, *et al.* Caring for Aged Dementia Care Resident Study (CADRES) of person-centred care, dementia-care mapping, and usual care in dementia: a cluster-randomised trial.[Erratum appears in Lancet Neurol. 2009 May;8(5):419]. *Lancet neurol.* Apr
Apr 2009;8(4):317-325.
37. Chenoweth L, *et al.* Study Protocol of a Randomised Controlled Group Trial of Client and Care Outcomes in the Residential Dementia Care Setting. *Worldviews on Evidence-based Nursing* 2010:doi: 10.1111/j.1741-6787.2010.00204.x.
38. Mitchell GK, *et al.* Do case conferences between general practitioners and specialist palliative care services improve quality of life? A randomised controlled trial (ISRCTN 52269003). *Palliat Med.* Dec 2008;22(8):904-912.
39. Abernethy AP, et al. Individualised multi-disciplinary case conferences: can they be practically added to specialist palliative care? *8th Australian Palliative Care Conference*. Sydney2007.
40. Abernethy AP, *et al.* A pragmatic 2 x 2 x 2 factorial cluster randomized controlled trial of educational outreach visiting and case conferencing in palliative care-methodology of the Palliative Care Trial [ISRCTN 81117481]. *Contemp Clin Trials.* Feb 2006;27(1):83-100.
41. Shelby-James TM, *et al.* Promoting patient centred palliative care through case conferencing. *Aust Fam Physician.* 2007;36(11):961-964.
42. Mid North Coast Division of General Practice. *Mid North Coast Rural Palliative Care Project: Final report.* Coffs Harbour: MNCDGP;2006.
43. Mid North Coast (NSW) Division of General Practice (MNCDGP), Coffs Harbour. *Toolkit: Creating a Multi-Disciplinary Team Approach to Care Planning In Residential Aged Care*

Facilities 2nd Edition. Coffs Harbour: MNCDGP: Rural Palliative Care Project, Aged Care GP Panels Initiative and Integrated Network Palliative Care Project;2007.

44. Halcomb EJ, *et al.* Perceptions of multidisciplinary case conferencing in residential aged care facilities. *Aust Health Rev.* Nov 2009;33(4):566-571.

45. Craig P, *et al.* Developing and evaluating complex interventions: the new Medical Research Council guidance. *Bmj.* 2008;337:a1655. Also available open-source online at www.mrc.ac.uk/complexinterventionsguidance.

46. National Institute for Health and Clinical Excellence. *Behaviour Change at Population, Community and Individual Levels. NICE Public Health Guidance.* London: NICE;2007.

47. Department of Health and Ageing. Aged Care Funding Instrument (ACFI). 2010; http://www.health.gov.au/internet/main/publishing.nsf/Content/ageing-acfi-factsheets.htm. Accessed Dec 12th, 2010.

48. Clayton JM, *et al.* Clinical practice guidelines for communicating prognosis and end-of-life issues with adults in the advanced stages of a life-limiting illness, and their caregivers. *Medical Journal of Australia.* 2007;186(12):S77- 108.

49. Rigby J, *et al.* Review: what evidence is there about the specific environmental needs of older people who are near the end of life and are cared for in hospices or similar institutions? A literature review. *Palliat Med.* 2010 24(3):268-285.

50. Kitwood T, Bredin K. Towards a theory of dementia care: personhood and well-being. *Ageing Soc.* 1992;12:269-287.

51. O'Neil T. Adding families to the care team. Family members hold keys to person-centered care. *Health Prog.* Nov-Dec 2009;90(6):48-50.

52. Burke C, *et al. Personcentred environment and care tool* [PhD]. Australia, University of Technology Sydney n.d.

53. Eagar K, *et al.* The Australian Palliative Care Outcomes Collaboration (PCOC)--measuring the quality and outcomes of palliative care on a routine basis. *Aust Health Rev.* May 2010;34(2):186-192.

54. Bellelli G, *et al.* The Bedford Alzheimer Nursing Severity scale for the severely demented: validation study. *Alzheimer Dis Assoc Disord.* Jun 1997;11(2):71-77.

55. Mitchell SL, *et al.* Advanced dementia research in the nursing home: the CASCADE study. *Alzheimer Dis Assoc Disord.* Jul-Sep 2006;20(3):166-175.

56. Pope C, Mays N, eds. *Qualitative Research in Health Care.* London: BMJ 2000.

57. Volicer L, *et al.* Scales for evaluation of End-of-Life Care in Dementia. *Alzheimer Dis Assoc Disord.* Oct-Dec 2001;15(4):194-200.

58. Kiely DK, *et al.* The Validity and Reliability of Scales for the Evaluation of End-of-Life Care in Advanced Dementia. *SO - Alzheimer Disease & Associated Disorders.* 2006;20(3):176-181.

59. Parker D, Hodgkinson B. A comparison of palliative care outcome measures used to assess the quality of palliative care provided in long-term care facilities: a systematic review. *Pall Med.* 2010;25(1):5-20.

60. van der Steen JT, *et al.* Evaluations of end of life with dementia by families in Dutch and U.S. nursing homes. *Int Psychogeriatr.* 2009;21(2):321-329.

61. Weiner MF, *et al.* The Quality of Life in Late-stage Dementia (QUALID) Scale. *J Am Med Dir Assoc.* 2000;1:114-116.

62. Smith SC, *et al.* Development of a new measure of health-related quality of life for people with dementia: DEMQOL. *Psychol Med.* May 2007;37(5):737-746.

63. Smith SC, *et al.* Measurement of health-related quality of life for people with dementia: development of a new instrument (DEMQOL) and an evaluation of current methodology. *Health Technology Assessment.* 2005;9(10):1-93,iii-iv.

64. Rowen D, *et al.* Estimating preference-based single index measures for dementia using DEMQOL and DEMQOL-Proxy. *Value in Health.* 2012;15:346-356.

65. Kind P. The EuroQoL instrument: An index of health-related quality of life. In: Spilker B, ed. *Quality of life and pharmacoeconomics in clinical trials*. 2nd ed. Philadelphia: Lipincott-Raven; 1996:191-201.

66. Hounsome N, *et al.* EQ-5D as a quality of life measure in people with dementia and their carers: evidence and key issues. *Value in Health.* Mar-Apr 2011;14(2):390-399.

67. Long CO, *et al.* Development of the questionnaire on palliative care for advanced dementia (qPAD). *Am J Alzheimers Dis Other Demen.* Nov 2012;27(7):537-543.

68. Reisberg B. Functional assessment staging (FAST). *Psychopharmacol Bull.* 1988;24(4):653-659.

69. Abernethy A, *et al.* The Australia-modified Karnofsky Performance Status (AKPS) scale: a revised scale for contemporary palliative care clinical practice. *BMC Palliative Care.* 2005;4(7):http://www.biomedcentral.com/content/pdf/1472-1684X-1474-1477.pdf.

70. Coventry PA, *et al.* Prediction of appropriate timing of palliative care for older adults with non-malignant life-threatening disease: a systematic review. *Age Ageing.* May 2005;34(3):218-227.

71. Norman GR, *et al.* Interpretation of changes in health-related quality of life: the remarkable universality of half a standard deviation. *Med Care.* 2003;41:582-592.

72. Mitchell SL, *et al.* The Advanced Dementia Prognostic Tool: A risk score to estimate survival in nursing home residents with advanced dementia. *Journal of Pain and Symptom Management.* 2010;40(5):doi:10.1016/j.jpainsymman.2010.1002.1014.

73. Darwiche A. *Modeling and Reasoning With Bayesian Networks*. New York: Cambridge University Press; 2009.

74. Bolstad WM. *Introduction to Bayesian statistics*. Hoboken, N.J.: Wiley-Interscience; 2004.

75. van den Hout A, Matthews FE. Estimating dementia-free life expectancy for Parkinson's patients using Bayesian inference and microsimulation. *Biostatistics.* Oct 2009;10(4):729-743.

76. Chen R, Herskovits EH. Network analysis of mild cognitive impairment. *Neuroimage.* Feb 15 2006;29(4):1252-1259.

77. Australian Bureau of Statistics. *Causes of Death, Australia, 2010.* Canberra: ABS;2012. 3303.0.

78. Access Economics. *Dementia estimates and projections, NSW and its regions.* Canberra2005.

79. Access Economics. *The dementia epidemic: Economic impact and positive solutions for Australia.* Canberra: Alzheimer's Australia;2003.

80. Gutterman E, *et al.* Cost of Alzheimer's Disease and Related Dementia in Managed-Medicare. *Journ Am Geriatr Soc.* 1999;47:1065 -1071.

81. Productivity Commission. *Caring for Older Australians, Draft Inquiry Report.* Canberra2011.

Appendix 1: Summary of measures and their timing

Level	Variable	Measure	Base-line	3-monthly	4 wks post-resident death	Study end
Facility	Stable characteristics	Location; size (number of beds); private versus not-for-profit status; organisational affiliation; dementia-specific status; accommodation and amenities; management structure	x			
	Characteristics liable to change	Staff profile and turnover (resignations and new staff); Resident to staff ratio; number and proportion of residents with dementia; number of new admissions and deaths per month; involvement of GPs and other community services; use of agency staff; availability of therapy and recreation programs; and proportion of residents requiring complex care.	x	x		
	Person-centredness of care	Care and Activities, and Interpersonal Relationships and Interactions' domain of the Person-Centred Environment and Care Assessment Tool (PCECAT)		6 months and 18 months only		x
	Quality of end of life care	Rates of acute care episodes and length of stay (including ED presentations with admission and actual admission) for residents with advanced dementia; rates of non-palliative interventions, defined as ventilation, resuscitation, nasogastric/PEG feeding and non-palliative pharmacological treatments;	x	x		
	(In intervention RACFs)	PCPC and other staff attendance at training; PCPC and other staff learning outcomes (pre-/post-training and 6-month follow-				

Level	Variable	Measure	Base-line	3-monthly	4 wks post-resident death	Study end
	adherence / fidelity	up qPAD); CC numbers, triggers, duration and attendance; care plan content and adherence; quality of documentation; quality of CCs rated via observation.				
	(For economic evaluation) CC costs	Session time, travel time and distance travelled by attendees		x		
Staff	Characteristics	Self-reported age, gender, qualifications, position, time in position, time in RAC, dementia experience and previous staff training.	x			
	Knowledge and attitudes	the Palliative Care for Advanced Dementia (qPAD)	x	First 3-monthly visit (must be post-training for interventio n arm)		
		(Via semi-structured interview) Perceptions of PCC and palliative care for advanced dementia and the importance of multi-disciplinary and family involvement in care planning				x
	(In intervention RACFs)	Semi-structured interview				x

Level	Variable	Measure	Base-line	3-monthly	4 wks post-resident death	Study end
	perceptions of intervention usefulness and facilitators/barriers					
	(For economic evaluation in intervention arm only) training costs	Materials, trainer's time and opportunity cost of trainee time	At time of training			
Resident	Characteristics	age; gender; previous occupation; years of education; time since dementia diagnosis; length of stay in RAC; family members; number of visitors and frequency of visits; most recent Aged Care Funding Instrument (ACFI) report; food and fluid intake; body mass index (BMI); PCOC phase; and Bedford Alzheimer Nursing Severity (BANS) Scale	x			
	Nurse-rated QOL	QUALID and EQ-5D-5L	x	x		
	Family- and nurse-rated symptom management	CAD-EOLD, SM-EOLD			x	

Level	Variable	Measure	Base-line	3-monthly	4 wks post-resident death	Study end
	and comfort at EOL					
	Other indicators of quality of end of life care	Rates of inappropriate acute care episodes, as judged again by an expert review of 10% of admissions; rates of inappropriate, non-palliative interventions		x		
	(For economic evaluation) routine healthcare costs	Use of GPs (for case conferences or other), specialists and pharmaceuticals		x		
Family	Characteristics	Self-reported relationship to resident, age, gender, education, occupation, dependents, frequency/duration of visits, person responsible status and prior involvement in decision-making	x			
	Satisfaction with EOL care for participating residents	SWC–EOLD			x	

Appendix 2: CONSORT CHECKLIST

SORT 2010 checklist of information to include when reporting a randomised trial*

Section/Topic	Item No	Checklist item
**Title and abstract**		
	1a	Identification as a randomised trial in the title
	1b	Structured summary of trial design, methods, results, and conclusions (for specific guidance see CONSORT for abstracts)

Cluster randomised controlled trial of facilitated case conferencing versus usual care for improving end of life outcomes in aged care residents with advanced dementia and their families.

IDEAL Project: Improving Dementia End of life care At Local aged care facilities.

**Introduction**		
Background and objectives	2a	Scientific background and explanation of rationale
	2b	Specific objectives or hypotheses

Cluster randomised trial addition: Scientific background and explanation of rationale, *including the rationale for using a cluster design*
Specific objectives and hypotheses *and whether they pertain to the individual level, the cluster level, or both*

Background
Dementia is a terminal disease. Care for people with advanced dementia requires a palliative approach that is targeted to the illness trajectory and tailored to the needs of each individual and his/her family. Currently, the quality of care in residential aged care (RAC) is compromised by lack of staff expertise and poor communication between staff, family and health professionals. Residents suffer unnecessary hospitalisations and aggressive treatments, while symptoms often go unmanaged. Facilitated case conferencing (FCC) is an approach that brings together RAC staff, health professionals and families to plan person-centred management based on best practice. FCC has improved outcomes in other palliative settings but evidence is lacking for RAC residents with advanced dementia. A cluster RCT design was chosen to minimise contamination between arms when taking a systems-based approach to intervention.

Objectives

This phase III cluster RCT aims to: 1) compare the efficacy of FCC with Usual care in improving end of life (EOL) outcomes for residents with advanced dementia living in RAC; 2) provide insights into facility- and staff-related processes influencing the implementation and sustainability of FCC; and 3) evaluate the cost-effectiveness of FCC versus Usual care.

Hypotheses

Primary hypothesis (Aim 1):
Compared with Usual care, FCC for residents with advanced dementia will achieve better family-rated EOL outcomes as defined by: a) better symptom-related comfort in the last 7 days of life; b) more effective symptom management over the last 90 days of life; c) greater family satisfaction with care over the last 90 days of the resident's life.

Hypothesis 2 (Aim 2):
Compared with Usual care, FCC for residents with advanced dementia will achieve:
a) better nurse-rated symptom-related comfort over the last 7 days of life and symptom management over the last 90 days of life;
b) higher scores on resident quality of life (QOL) at last measurement prior to resident death;
c) a palliative approach to medical care of residents with advanced dementia, as evidenced by reduced acute care episodes, non-palliative interventions and adverse incidents at the facility level;
d) a person-centred approach by RACF staff to all aspects of care;
e) improvements in RAC staff attitudes to, knowledge of and confidence in providing palliative care to residents with advanced dementia and dementia care more generally.
f) improvements in family QOL.

Hypothesis 3 (Aim 3):
Compared with Usual care, FCC for residents with advanced dementia will EITHER result in an overall reduction in health care costs with no loss to resident health OR be cost-effective in terms of health gained at acceptable additional cost.

**Methods**		
Trial design	3a	Description of trial design (such as parallel, factorial) including allocation ratio
	3b	Important changes to methods after trial commencement (such as eligibility criteria), with reasons

Cluster randomised trial addition: How participants were allocated to interventions (e.g. random allocation, randomised, or randomly assigned), *specifying that allocation was based on clusters*

The proposed research will use a phase III parallel cluster RCT design, conducted in 6 stages over 3 years, with a 1:1 RACF allocation to either FCC or UC. Activities for each stage will be as follows:

1. *Set-up*: Project staff recruitment; application for ethics approval; identification of eligible RACFs and randomised approach; RACF level consents; RACF staff consents; recruitment and training of research staff (including inter-rater reliability).
2. *Pre-test:* Collection of resident/family consent; baseline data collection at RACF, dyad and staff levels; identification, selection and appointment of “Palliative Care Planning Coordinators” (PCPCs) at each Intervention RACFs.
3. *Intervention (training):* The responsibility for FCC will be the role of PCPCs - with continuous training for other RAC staff. This stage will include Palliative care planning coordinator (PCPC) baseline data collection and training by research staff; PCPC post-training evaluation of learning outcomes; ongoing support of case conferencing (CC) & PCPC training of other staff in the RACF:
4. *Delivery of FCC and follow-up* - Ongoing recruitment of any newly admitted residents who meet eligibility criteria until 27 months (to allow 6 months follow-up period for outcomes); organisation of CC at relevant trigger points (see below), 3-monthly collection, entering and checking of process and outcomes data at all sites; interviews with family two months following resident death.
5. *Final follow-up*: Final data collection from resident/family dyads and follow-up of residents with advanced dementia who were enrolled late in the project period; exit interviews with PCPCs and random samples of families, RAC staff and community health professionals involved in care at each RACF.
6. *Close-out*: Data checking, analysis and reporting; feedback to participants; analysis and reporting.

Participants	4a	Eligibility criteria for participants *and clusters*
	4b	Settings and locations where the data were collected

Eligibility criteria for clusters/RACF:

- size (≥100 beds)
- ≥50% residents with dementia
- identified as high care.

Eligibility criteria for residents:

- a diagnosis of dementia & advanced disease as determined by Functional Assessment Staging Tool (FAST; [68]) in dementia ≥6a,
- stable for 1 month according to RACF staff;
- Australia–modified Karnofsky Performance Status (AKPS; [69]) of ≤50;
- availability of a person legally responsible to give consent on their behalf;
- informed consent from a family member or other who knows the resident well & will participate as the second member of the dyad.

The FAST has been chosen because of its utility in the advanced stages of dementia; a FAST stage 7c & functional dependency (measured here by AKPS) is predictive of <6 months survival.

Eligibility for family/friend "informant" (ideally the person legally responsible):

- visits the resident at least once a fortnight;
- knew the resident prior to their dementia diagnosis;
- is willing to be involved in decisions about the resident's care;
- English proficiency sufficient to complete outcome measures (below).

RAC staff:
all permanent nursing/personal care staff.

Other health professionals (e.g. GPs) involved in case conferencing for participating residents will also be eligible to participate.

Settings and locations where the data were collected

Brisbane and Sydney, metropolitan

Interventions	5	The interventions for each group with sufficient details to allow replication, including how and when they were actually administered

Cluster randomised trial addition: Precise details of the interventions intended for each group, *whether they pertain to the individual level, the cluster level, or both.*

INTERVENTION:
We will use a train-the-trainer model, whereby a Palliative Care Planning Coordinator (PCPC) is trained and then supported to train other nursing and personal care staff at each RACF.

Palliative Care Planning Coordinators (PCPCs): RACFs randomised to the Intervention arm will be invited to discuss allocation of PCPC responsibility best-suited to local conditions. The PCPC will be the team leader in each RACF responsible for implementing the CC model as per the protocol and providing ongoing education and mentoring to other RACF staff (see below). Our pilot experience suggests the role will best suit a permanent, full-time, senior nurse. (S)he will require communication skills and clinical expertise appropriate to dementia and palliative care. PCPCs will be expected to commit 0.5FTE to the role, paid at their usual rate. Selection will be via interview and, once appointed, relevant duties will be added to the staff member's job description. PCPCs will attend one week full time equivalent of training at a central location. To assess learning outcomes, PCPCs will complete pre- and post training administrations of the Palliative Care for Advanced Dementia (qPAD), a 35-item tool assessing health professionals' knowledge of advanced dementia care needs and attitudes to providing related care.

PCPC training in advanced dementia-specific FCC and person-centred, palliative/EOL care: PCPCs will learn to *identify 'triggers' that warrant initiation of a FCC,* including: admission to RACF; return to RACF following discharge from acute care or Emergency Department (ED) presentation; increase in falls; change in clinical status; new/worsening symptoms; poor appetite; poor skin integrity; at the annual management plan review in conjunction with the Aged Care Funding Instrument [ACFI]; receipt of a complaint; family disagreement about care; family distress.

Where a trigger for FCC has been identified, the point of disease progression will be mapped onto relevant domains to indicate whether survival time will likely be measured in months, weeks or days. The "map" for each resident will highlight issues for discussion at CC, including pre-emptive planning for predicted deterioration. The PCPC will receive training in organising CCs and receive *specific guidance on when and how to initiate advance care planning regarding do-not-resuscitate (DNR) and do-not-hospitalise (DNH) orders and artificial nutrition/hydration*.

Training in facilitating general practitioner involvement: Strategies will include using a direct telephone approach with an offer of a follow-up visit to discuss, assistance with obtaining remuneration for time spent, and ensuring that FCCs are as time-efficient as possible. Wherever possible, GPs will be given advance notice of FCCs and asked to book them in as appointments. FCCs will be arranged at times to suit GPs (e.g., lunch times), and GPs will be given the opportunity to join by teleconference only for those CC contents most relevant to them (e.g., symptom management). These approaches have led to impressive GP attendance rates in a previous RCT by the team.

Palliative environment: Finally, training will assist RACF staff to provide a "palliative" space (an area which provides the resident and family a quiet, homelike private environment 24 hours per day.

PCC training will make use of experiential and adult learning approaches, guidance on how family members can be involved as much as possible in decision-making about care planning, implementation and monitoring, and support reconceptualisation from a "service focus" to person-centred focus.

Train-the-trainer training will provide PCPCs with the skills and resources to deliver one-day training and mentoring in advanced dementia-specific CC and PCC palliative care to other nurses and care staff. Ongoing support in the Intervention arm will be provided following initial training and for the rest of the study period. The team will liaise monthly with each PCPC to discuss progress and difficulties and plan support of staff training and CCs where monitoring data (see below) indicate this is required.

<u>USUAL CARE</u>:
There will be no additional education, training or support provided to Control RACFs but there will also be no restriction on service-provider education and training where this is current practice. The level, duration and type of such initiatives will be documented.

Outcomes	6a	Completely defined pre-specified primary and secondary outcome measures, including how and when they were assessed
	6b	Any changes to trial outcomes after the trial commenced, with reasons

Cluster randomised trial addition: Report clearly defined primary and secondary outcome measures, *whether they pertain to the individual level, the cluster level, or both*, and, when applicable, any methods used to enhance the quality of measurements (e.g. multiple observations, training of assessors)

Primary:
Family-rated EOL outcomes will be measured using the End of Life in Dementia (EOLD) Scales (Volicer et al., 2001).

The EOLD Scales will be used to measure symptom-related comfort during the last seven days of life (CAD-EOLD), symptom management in the last 90 days of life (SM-EOLD) and family satisfaction with care during the last 90 days of life (SWC-EOLD). While the CAD-EOLD and SM-EOLD have been validated for rating either by family or nurses, family ratings will be our primary outcome because family perceptions of EOL suffering and its management are important outcomes for palliative care and our intervention's staff focus may lead to response bias in nurse ratings. All scales will be rated 4 weeks following resident death.

Secondary:

a. Nurse-rated symptom-related comfort and symptom management will be measured using the CAD-EOLD and SM-EOLD Scales, and rated 4 weeks following residents' death by the nurse who has provided most care in the last 7 days and 90 days of life respectively.
b. Nurse-rated resident QOL will be measured second weekly using the Quality of Life in Late-stage Dementia (QUALID) Scale [61]and EQ-5D-5L [66] for economic evaluation purposes.
c. A palliative approach to care at the facility level will be measured using the following indices:
   - rates of acute care episodes and length of stay (including ED presentations with admission and actual admission) for residents with advanced dementia; and
   - rates of inappropriate acute care episodes, as judged again by an expert review of 10% of admissions at each RACF. Documents reviewed will be nursing records, transfer letters, discharge summaries and medical records written on admission and discharge;
   - rates of non-palliative interventions, defined as ventilation, resuscitation, nasogastric/PEG feeding and non-palliative pharmacological treatments;
   - rates of inappropriate, non-palliative interventions, as judged by expert review of nursing records;
   - rates of adverse events, identified via a review of nursing records and incident charts and coded as: falls with/without injury, skin tears, injuries during care and medication incidents; and number/type of complaints from families (e.g. quality of care).
d. RACF person-centred approach to care will be measured by the 'Care & Activities, & Interpersonal Relationships & Interactions' domain of the Person-Centred Environment & Care Assessment Tool (PCECAT).
e. RAC staff's attitudes to and knowledge of providing palliative/EOL care to residents with advanced dementia will be evaluated using the 35-item Palliative Care for Advanced Dementia (qPAD.[67]

Sample size	7a	How sample size was determined
	7b	When applicable, explanation of any interim analyses and stopping guidelines

Cluster randomised trial addition: How *total* sample size was determined *(including method of calculation, number of clusters, cluster size, a coefficient of intracluster correlation (ICC or k), and an indication of its uncertainty*) and, when applicable, explanation of any interim analyses and stopping rules

In the absence of established minimal clinically important differences on the EOLD Scales, we will follow a common rule-of-thumb and assume 0.5 standard deviation (SD) [71]. Sample size has been based on the EOLD scale with the highest intra-cluster correlation (ICC) (i.e., requiring the greatest number of clusters). To detect a change in CAD-EOLD of half a standard deviation (a change in score of at least 3), with a two-sided 5% significance level, power of 80% and an intracluster correlation coefficient of 0.05 (estimated from unpublished data sourced from Dutch RAC facilities), a sample size of 8 clusters per group (16 in total), with 15 residents per cluster, was necessary. Given an anticipated dropout rate (at the resident level) of 10%, a recruitment sample of 272 residents (approximately 17 per site) would be needed for a final sample of 240 residents at 8 facilities. An interim check conducted on blinded data as of 6 months data collection will be performed to assess whether any of these assumptions requires adjustment and a re-estimation of sample size is necessary. The feasibility of accruing 17 residents with advanced dementia per RACF with a life expectancy less than the study period (<18 months) is based on: 1)

a review of death data at Hammond Care RACFs, and 2) research showing that 40% of people admitted to RAC with advanced dementia die within 1 year and our eligibility criteria predict further decreases in life expectancy.[70,72]

Randomisation:		
Sequence generation	8a	Method used to generate the random allocation sequence
	8b	Type of randomisation; details of any restriction (such as blocking and block size, stratification, matching)
Allocation concealment mechanism	9	Mechanism used to implement the random allocation sequence (specifying that allocation was based on clusters rather than individuals), and clarifying whether the sequence was concealed until interventions were assigned
Implementation	10	Who generated the random allocation sequence, who enrolled participants, and who assigned participants to interventions/their groups

Block randomisation will occur using two stratification factors (dementia specific unit or not; part of organisation or stand alone facility) using a computer generated random allocation sequence developed by the study statistician. This method will facilitate balance in the allocation of RACFs to the FCC and UC groups. The size of the blocks will vary randomly between two, four and six. Facilities will be enrolled prior to assignment. Enrolment and assignment will be undertaken by the project manager who will in any case need to be unblinded in order to liaise with facilities in the FCC arm regarding implementation of the intervention. The random allocation sequence will be concealed until facilities are assigned.

Blinding	11a	If done, who was blinded after assignment to interventions (for example, participants, care providers, those assessing outcomes) and how
	11b	If relevant, description of the similarity of interventions

Cluster randomised trial addition: Whether participants, those administering the interventions, and those assessing the outcomes were blinded to group assignment. If done, how the success of blinding was evaluated.

In order to avoid selection bias, recruitment of patients was by research assistants masked to the cluster allocation. The research assistants will be blinded to the purpose of the study, and each will visit only RACFs in either the Intervention or Control arm. The statistician allocating sites to FCC or UC groups will be blind to the identity of the RACFs. Project managers will need to be unblinded in order to liaise with PCPCs at Intervention RACFs; they will not collect process or outcome data after baseline.

Statistical methods	12a	Statistical methods used to compare groups for primary outcome(s) *indicating how clustering was taken into account*
	12b	Methods for additional analyses, such as subgroup analyses and adjusted analyses

<u>Aim 1 – Efficacy</u>: Primary and secondary analyses will be performed on an intention to treat (ITT) basis. Descriptive statistics will be used to compare characteristics at individual- and facility-levels in each group at baseline, accounting for the clustering effect in the former. Mixed or 'multilevel' modelling will be used to determine the effects of FCC and Usual care on primary and individual-level secondary outcomes (i.e. all secondary hypotheses except d). Mixed models allow adjustment for both individual (i.e. resident, family or staff) and cluster-level covariates as well as adjustment for the inherent correlation within clusters. Cluster level analyses will be implemented to determine the effect of the intervention on facility-level outcomes (i.e. hypothesis 2d). Analyses will be weighted by cluster size as required. Results will be interpreted and generalised accordingly.

<u>Aim 2 – Process and sustainability</u>: Bayesian inference networks analysis (BINA) will be used to further model the influence of resident, family, staff, RACF and intervention variables on processes and outcomes. This analysis will overcome two limitations of traditional statistical analyses, namely: 1) inability to detect intervention effects due to smaller than expected effect size or heterogeneity in participants or facilities, and 2) relevance of results only to groups with certain 'average' characteristics that cannot be easily translated to each individual. BINA overcomes these problems by linking individuals' characteristics (e.g., severity of dementia) and events (e.g., change in clinical status) to estimate likelihoods of future improvement or deterioration. These models enable initial assumptions regarding efficacy to be modified and probabilities recalculated to accommodate new observations. Network analysis will also be used to model relationships between family and staff EOLD Scale ratings in each arm to provide insight into potential direction and degree of response bias. BINA is widely applied in information technology (e.g., the *Google* search engine) but use in health research and dementia care is only just emerging and observational rather than evaluative data have been used. BINA will be carried out by Dr Victor Vickland, from the Dementia Collaborative Research Centre (DCRC) (UNSW). Dr Vickland is CIA on an NHMRC-funded project using BINA to develop computer-assisted clinical guidelines of anxiety and depression management. *Qualitative analysis* of interview data will be supervised by CIs Chenoweth, Beattie and Phillips and will use standard coding and classifying techniques and N-Vivo software.

<u>Aim 3 - Economic evaluation</u>: The incremental benefit of FCC will be calculated in terms of QALYs gained. The cost of the intervention will be measured along with any cost-savings due to avoided healthcare utilisation. Results will be presented in terms of the incremental cost-effectiveness ratio (ICER). Many of these model parameters will not be powered for statistical significance. Therefore, mean estimates of resource utilisation will be used and confidence intervals will be generated by boot-strapping the data. Uncertainty will be explored using probabilistic sensitivity analysis.
